# Generational differences in mental health and drivers of youth mental health decline in Australia and the United Kingdom

**DOI:** 10.3389/fpubh.2026.1844747

**Published:** 2026-07-14

**Authors:** Samitha Udayanga

**Affiliations:** Bremen International Graduate School of Social Sciences, University of Bremen, Bremen, Germany

**Keywords:** age-period-cohort, Gen Z, mental health, Millennials, social networks

## Abstract

**Background:**

The sharp decline in mental health among younger generations has become a key global health crisis of the twenty-first century. While often linked to short-term life-cycle stages or common period effects, emerging evidence indicates distinct “generational scarring” among Millennials and Gen Z (Generation Z). The causes of this decline in mental health are still debated, with theories ranging from economic insecurity to social fragmentation.

**Objective:**

The present study aims to disentangle Age, Period, and Cohort (APC) effects to determine whether the youth mental health crisis is a function of developmental stage or structural cohort disadvantage. In particular, it investigates the differential roles of economic capital, social conditions, and social networks in shaping mental health trajectories in the United Kingdom and Australia.

**Methods:**

We analyzed longitudinal data from the Understanding Society survey (UK) and the Household, Income and Labor Dynamics in Australia (HILDA) survey (Australia). We employed generalized additive models (GAMs) to visualize trajectories and Hierarchical Age Period and Cohort models to estimate what explains generational differences in mental health.

**Results:**

Cohort variance exceeded period variance in both countries, indicating that youth mental health decline is primarily generational rather than period driven. Gen Z entered adulthood with significantly lower mental health baselines than earlier cohorts in both countries, although Australian Gen Z showed a U-shaped recovery after age 20 while UK Gen Z showed no recovery. In the United Kingdom, the Gen Z deficit was almost fully mediated by perceived social isolation, indicating a crisis of connection, whereas the Millennial deficit persisted net of all economic and social controls, consistent with lasting scarring from the post-2008 austerity period. In Australia, standard economic, family, and social support variables failed to resolve the generational mental health gap.

**Conclusion:**

The drivers of youth mental health decline are structurally distinct between Australia and the UK. The United Kingdom requires investment in social infrastructure and community repair, while the persistent Millennial scar signals lasting damage from formative-years adversity. In Australia, the crisis is rooted in restricted occupational agency, requiring labor market reforms that enhance autonomy for younger workers. Public health responses must move beyond a uniform medical model of individual pathology toward context-specific structural interventions that treat social connection and agency as population-level determinants of mental health.

## Introduction

The decline in mental health among younger generations has become a defining public health crisis of the twenty-first century (1, 2). The World Health Organization (WHO) defines mental health as a condition of psychological well-being that allows individuals to manage stress, fulfill their potential, engage in learning and work effectively, and contribute to their communities (3). Across many developed countries, indicators of psychological distress, anxiety, and depressive symptoms have increased sharply over the last decade, with the steepest decline observed among adolescents and young adults (4, 5). While mental health has generally shown life-course variation, often following a U-shaped curve in which distress peaks in early adulthood and old age (6), recent surveillance data suggest a fundamental structural shift (7). The midlife crisis of previous decades appears to be shifting downward, morphing into a chronic quarter-life crisis for Millennials (born 1980–1994) and Gen Z (born 1995 onwards) (8, 9).

Whether this concentration of mental health decline among the young reflects a temporary or structural problem depends on its temporal sources. In this context, a central epidemiological challenge lies in disentangling Age, Period, and Cohort (APC) effects on mental health (10, 11). Several methodological approaches have been proposed for this purpose (12–14), and studies report mixed findings. Some emphasize exogenous shocks as drivers of mental health decline, while others highlight age and cohort differences as key determinants (11). Although these studies have examined age, period, or cohort effects on mental health, the underlying causes of such effects have rarely been investigated.

The etiology of mental health decline remains a global concern, with a major focus on neurological causes (15, 16), and sociocultural determinants (17) within the one health framework (18). While the severe burden of mental health problems among children and older adults has received considerable attention in such studies (19–21), the causes of the youth mental health crisis and generational differences are still emerging from recent studies (11, 22, 23). Since different cohorts undergo different exogenous experiences, their mental health trajectories may also diverge, as in the case with physical health (24, 25). For example, Gen Z and Millennials are deeply aware of and engaged with global issues, consuming more information about uncertainty than other content types and expressing willingness to shape their societies. UNICEF thus reports that these younger generations are more prone to mental health problems than older generations (26). Distinguishing these mechanisms is important as it has profound policy implications.

In the present study, we leverage longitudinal data from two comparable yet distinct welfare regimes (Australia and the United Kingdom) to disentangle these APC effects and interrogate the social determinants of youth distress. Importantly, we test three hypotheses: the economic precarity hypothesis (employment and income), the life-course buffer hypothesis (improving education and family building), and the social connectedness hypothesis (social networks).

Our study makes three important contributions. First, it provides a comparative APC decomposition of mental health for the United Kingdom and Australia using country-based longitudinal panels, extending the hierarchical APC framework to panel data with individual-level corrections for unobserved heterogeneity. Second, it moves from documenting generational decline to adjudicating between mechanisms, testing whether the cohort penalty is a crisis of material deprivation, of delayed adulthood, or of social fragmentation. Third, by comparing how the same mechanisms behave in two similar welfare regimes, it identifies whether policy lessons on youth mental health can be transferred across countries.

## Literature review and theoretical basis

### Youth mental health in the UK and Australia

Globally, approximately one in five individuals lives with a mental health condition at any given time, a burden that was further exacerbated following the onset of the COVID-19 pandemic (3). Moitra et al. (27) observed that mental illness has increased globally, disproportionately affecting vulnerable populations, while treatment coverage remains low. As the WHO consistently notes (3), despite major global focus on mental health, it continues to be neglected in some regions due to stigma (28). Even in advanced economies where mental health has received considerable attention in public health policy, it remains less discussed at the population level. For example, existing studies have documented deteriorating mental health among young people in various high-income countries, including Australia (10), and the United Kingdom (29).

The selection of the United Kingdom and Australia for our analysis is grounded in a ‘most similar systems’ research design (30). Both countries are high-income, liberal welfare regimes characterized by similar institutional frameworks, cultural baselines, and individualistic labor markets. This foundational similarity acts as a macro-level control, providing a robust baseline from which to examine their diverging trajectories over the past two decades. However, the two countries have navigated clearly different contemporary structural shocks. The UK has experienced over a decade of severe economic austerity, significant reductions in community infrastructure, and the social fracture associated with Brexit, creating a landscape ripe for social fragmentation (31). Conversely, Australia largely bypassed the severe recessionary impacts of the Global Financial Crisis but has increasingly become defined by its exposure to extreme macro-environmental stressors, such as unprecedented bushfires and climate volatility, alongside an exceptionally rigid and financialized housing market (32). By comparing these two contexts using their respective premier longitudinal datasets (Understanding Society and HILDA), this study disentangles whether the generational scaring of youth mental health is a universal feature of late modernity or a highly contextual response to specific social pathologies.

In Australia, the prevalence of mental ill-health remains high despite the country’s overall health system stability (33). According to national surveillance data, 22% of the population aged 16 to 85 experienced a mental disorder in the last 12 months, while 43% (approximately 8.5 million people) have experienced a mental illness at some point in their lifetime (34). These burdens are not gender neutral; women bear a significantly greater burden of distress compared to men, revealing gender-specific vulnerabilities in life trajectories (33). Furthermore, a concerning paradox exists where mental health among young people continues to deteriorate despite broad improvements in social and economic development (10). Botha et al. (10) and Burns et al. (35), utilizing longitudinal data from the HILDA survey, emphasized that younger generations are experiencing worsening mental health trajectories, including increasing depressive symptoms that correlate with rising suicide risks. They highlight the urgent need for further cross-country investigation into the trajectory differences and underlying drivers of this depreciation.

The United Kingdom presents a similar profile, where staggering prevalence rates highlight a deep-seated crisis (36). Estimates suggest that one in four people experience symptoms of mental health problems annually. By 2024, approximately 20% of the population was found to be experiencing anxiety disorders or depression, with young people placed at a significant disadvantage (36). Unlike Australia, the UK context is heavily characterized by widening socioeconomic inequalities and ethnicity-based discrimination (37). Structural disparities are evident in clinical outcomes; for instance, Black or Black British individuals exhibit significantly higher rates of psychotic disorders (3.2%) compared to White individuals (0.3%) (38). Longitudinal comparisons reveal a sharp deterioration for youth: compared to 1993, young people are now significantly more likely to experience common mental health conditions, with prevalence rising to 25.8%. At the same time, suicide rates for young people have increased to 11.4 deaths per 100,000 persons (38).

### The age-period-cohort identification problem

While recent studies have sought to decipher the causes of mental health depreciation, a decomposition of Age, Period, and Cohort (APC) effects remains absent in the UK context compared to Australia (10, 11, 37). This paper addresses this gap by conducting a comparative APC analysis to determine whether these trends are driven by life-cycle stages or by specific generational scarring. The interpretation is done separately for the two countries, as direct comparison is challenging owing to measurement variance.

While psychological distress has historically followed a U-shaped trajectory over the life course, peaking in middle age (6), recent surveillance data suggests a linearization of this trend, with morbidity increasingly concentrated among adolescents and young adults (2). Conventional analyses often attribute this to “Period effects,” universal stressors such as the Global Financial Crisis or the COVID-19 pandemic that affect all age groups simultaneously (39). However, emerging evidence suggests that specific birth cohorts, particularly Millennials and Gen Z, exhibit a residual ‘scarring’ effect that persists regardless of age or the current period (2, 8, 10).

Previous studies have widely used the APC framework to understand population mental health, though they vary considerably in how it is operationalized. Some treat age as a proxy for developmental or life-course processes without distinguishing biological maturation from social role transitions (40). Others collapse birth cohorts into broad generational categories (e.g., ‘Gen Z’ and ‘Millennials’), focusing mainly on formative years through which certain time specific factors imprint on the behavior of persons (10). Period effects are often inferred from aggregate time trends without linking them to specific exogenous events or policy changes (14).

Beyond APC effects, mental health outcomes vary by gender, geographic location, socioeconomic status, and other sociocultural factors (41). These heterogeneous patterns have been documented across children, adults, and older adults, reinforcing the need for multidimensional explanations (27, 41). However, APC identification is central to public health research, as it clarifies whether observed mental health trends reflect developmental processes, generational differences, or broader societal and environmental conditions. Various methodological approaches have been proposed to address the well-known identification problem inherent in APC models (23, 42, 43), bringing about complementary but sometimes conflicting findings.

For instance, some studies attribute rising psychological distress among young people primarily to cohort effects, emphasizing formative experiences such as smartphone adoption and changing parenting norms (4). Others emphasize period effects, pointing to economic recessions, labor market precarity, or pandemics as drivers affecting all age groups (10, 23, 41, 44). Still certain studies highlight age effects, suggesting that observed trends reflect normative developmental challenges rather than secular change (40). Moreover, some studies also highlight the equal importance of all age, period and cohort effects on mental health (11).

The fundamental identification problem in APC analysis, where age, period, and cohort are linearly dependent, remains a persistent challenge. A number of solutions have been proposed for this problem, including constrained estimators (45), hierarchical APC models (12), and bounding analysis (46). Each approach relies on different assumptions that are often difficult to verify empirically, and simulation studies have demonstrated that these methods can bring conflicting conclusions from identical data (14). Recent advances using longitudinal panel data offer a partial resolution by tracking individuals over time, thereby separating within-person age effects from between-cohort differences (10). We follow the cross classified random effects models together with longitudinal data following Yang et al. (12) and Both et al. (10) However, panel attrition and selective mortality can introduce bias, particularly for older cohorts or individuals with severe mental health problems. This is one of the limitations in the present study.

### Mechanisms of generation mental health difference: three hypotheses

Prior research has proposed several explanations for declining youth mental health, including increased social media use and loneliness (47); rising economic precarity (48); declining social connectedness (4); climate-related anxiety (49); and exposure to global issues and related information (26). All of these studies highlight the declining mental health among younger generations compared to older generations, yet structural factors associated with life course transitions have received less attention or interpretation, as they are often considered covariates, rather than main exposures. Importantly, these explanations are rarely integrated within an APC framework that distinguishes whether they operate as cohort-specific formative experiences, period-specific shocks, or age-graded life-course processes. Disentangling these mechanisms requires research designs that explicitly model APC effects alongside potential explanatory factors. Therefore, following Yang et al. (12), we use hierarchical APC method in the present study.

The first hypothesis posits that the mental health decline in younger cohorts is a function of material deprivation and exclusion from the ‘asset economy’ (50). Unlike the post-war cohorts who benefited from robust social mobility, expanding home ownership, and relatively stable employments, Millennials and Gen Z face a unique economic reality characterized by stagnant real wages and the ‘privatization of risk’ (51). Public health literature consistently demonstrates an association between socioeconomic status (SES) and mental health (51, 52); however, recent scholarship argues that economic stability is the more salient determinant for younger generations (53, 54). The transition to adulthood is largely dependent on access to employment opportunities. This indicates that the inability to build such assets and stabilize private economic life can be a key factor in the decline of mental health among younger groups.

The finding that younger generation including millennials and Gen Z experienced a decline in mental health can thus be explained in terms of economic precarity hypothesis, in which concerns for economic stability among younger generations can generate an existential insecurity leading to distress. Employment, and financial stability are central to ontological security of human beings as Giddens’ described (55), and when these are deviated from or inaccessible, individuals may experience heightened psychological distress. This precarity is particularly pronounced among younger cohorts who disproportionately occupy insecure employment arrangements, including zero-hours contracts, gig work, and fixed-term positions, particularly in the UK and Australia (56).

Virtanen et al. (57), in a meta-analysis of 27 studies, found that temporary employment was associated with elevated psychological morbidity compared to permanent employment. Benach et al. (58) extended this finding, demonstrating that not only unemployment but also precarious employment, characterized by instability, low wages, and limited rights, is associated with adverse mental health outcomes. Butterworth et al. (59), using Australian HILDA data, found that poor-quality employment was associated with mental health outcomes comparable to or worse than unemployment, suggesting that job quality, not merely employment status, is relevant for psychological wellbeing. While this socioeconomic status and mental health association is well-established across age groups (41), recent scholarship argues that economic stability operates differently for younger generations. Mainly, the transition to adulthood has become increasingly protracted and uncertain. Arnett’s (60) concept of ‘emerging adulthood’ describes a distinct developmental phase characterized by identity exploration and instability. When structural conditions, such as labor markets and low income, impede the achievement of traditional adult milestones (stable employment, and family formation), the psychological stability is likely to be disrupted.

*Hypothesis 1*: The generational mental health gap is primarily driven by material deprivation with regard to economic factors.

The second hypothesis posits that mental health decline in younger cohorts is driven not solely by economic precarity but by the decline of traditional structural buffers that historically facilitated psychological well-being during the transition to adulthood. These buffers, including marriage, parenthood, and the protective returns to education, have either been delayed, diminished, or transformed in ways that leave younger cohorts more exposed to psychosocial stressors. Beyond material assets, shifting social conditions and life-course milestones shape the mental health landscape of new generations (17). The ‘traditional markers’ of adulthood (such as marriage and parenthood) have historically served as protective buffers against psychological distress through the provision of emotional stability and social integration (61–63). However, demographic shifts have delayed these milestones for younger cohorts. While higher education was previously a guarantor of both mental health and overall well-being, the rapid expansion of university attendance has led to credential inflation. Recent studies suggest that for Gen Z, the protective effect of education may be diminishing, as it is being replaced by the stress of academic competition and the burden of student debt (64, 65).

In this regard, the life course theory highlights how the timing, sequencing, and social context of key transitions shape individual development, including leaving the parental home, completing education, entering stable employment, forming partnerships, and becoming a parent (66, 67). When these transitions are delayed, disordered, or rendered inaccessible, individuals are likely to experience role ambiguity and identity confusion, with consequences for mental well-being (68). Previous studies document that for example, marriage and sustained family life is associated with lower rates of depression and psychological distress through mechanisms including emotional support, economic pooling, and enhanced meaning and purpose (69).

However, recent research suggests that the protective effects of traditional adult transitions may be attenuating for younger cohorts. Extended periods without stable partnership coincide with rising loneliness among young adults, a well-established risk factor for mental health problems (47, 70). Similarly, delayed or involuntary childlessness driven by structural constraints may deprive individuals of meaning and social integration. In addition, educational expansion has further restructured the transition to adulthood by delaying labor market entry and household formation, leaving young people in a prolonged liminal status that, combined with uncertain post-graduation prospects, may contribute to identity confusion and future-oriented anxiety leading to decline in mental health (68).

*Hypothesis 2*: The generational mental health gap is driven by the delay or loss of traditional life-course buffers of adulthood.

The third hypothesis concerns the decline in the vitality of social networks (71). Social networks, defined by strong community ties, trust, and reciprocal support are well-established determinants of resilience and mental health (72). Although online social networking can also be supportive for mental health (73), recent studies show that the *quality* of social networks matters more than the mere presence of connections (4). For example, having someone to confide in and rely on when problems occur is associated with greater mental resilience (71). Accordingly, we focus on such productive relationships that emerge from meaningful and supportive interactions between individuals. Putnam’s (74) diagnosis of declining social cohesion has accelerated notably for younger generations. Despite being the most digitally connected cohort in history, Gen Z reports the highest prevalence of loneliness and social isolation (33, 75). This paradox is often explained by the displacement hypothesis, which argues that digital interaction has displaced the face-to-face contact necessary for genuine emotional regulation and support (73). If the generational mental health deficit is mediated by a decline in social support, it implies that the crisis is fundamentally one of social fragmentation rather than economic deprivation alone.

The social networking and stress-buffering explanation posits that social support protects mental health by providing emotional, informational, and instrumental resources that help individuals cope with stressors. For example, Acoba (76) shows that social support reduces perceived stress, which in turn reduces stress conditions and anxiety while improving positive affect. Longitudinal studies also demonstrate that individuals with stronger social ties exhibit lower rates of depression and anxiety, with effects operating through both main effects (the direct benefits of integration) and buffering effects (protection during stress) (77).

Along with these explanations, recent studies support generational declines in social connectedness. Twenge et al. (4) found that adolescents’ in-person social interaction declined substantially after 2012, coinciding with smartphone adoption. Moreover, young adults with high social media use reported greater perceived social isolation, suggesting digital connectivity does not substitute for face-to-face contact as recent studies documented (78, 79). Therefore, the decline in social connectedness and social network quality among younger generations can have a negative impact on mental health.

*Hypothesis 3*: The generational mental health gap, after accounting for economic and life-course factors, is driven by declining quality of social connectedness.

These three hypotheses represent integrated explanations for generational mental health decline mainly observed among younger cohorts (millennials and Gen Z). The economic precarity hypothesis, rooted in existential psychological security scholarship, locates the explanation in material conditions such as economic security. However, existential security emerging from structural economic circumstances does not function alone, and it is also associated with other life course circumstances, as the second hypothesis suggested. The life-course hypothesis emphasizes that biographical transitions carry intrinsic psychological significance in addition to existential security established through economic conditions. Marriage, education, and parenthood offer meaning, purpose, and identity consolidation that transcends economic pooling or social network expansion. From this perspective, cohort differences in mental health would persist even among economically secure young adults who have not achieved traditional milestones. The social connectedness hypothesis emphasizes the quality of interpersonal bonds as the fundamental determinant of mental health. From this perspective, economic security and role occupancy matter only insofar as they facilitate meaningful relationships. A young adult with stable employment and a partner but lacking intimate confidants would remain vulnerable to distress. All these three hypotheses posit an integrated explanation for possible reasons for youth mental health decline in comparison to older cohorts.

## Methods

### Data

Our study utilizes longitudinal data from two nationally representative household panel surveys: the Household, Income and Labor Dynamics in Australia (HILDA) survey (80) and the Understanding Society (UKHLS) survey for the United Kingdom (81). Both datasets provide granular, annual information on respondents’ mental health, socioeconomic status, and social networks. These two data sets have also been widely used in public health research. We used 22 waves of HILDA data spanning 2001 to 2023. HILDA is a household-based panel study that began with a national probability sample of Australian households. For the UK, we utilized data from *Understanding Society*, covering the period from 2009 to 2023. Observations with missing data for the dependent variable were deleted, and missing data on time-varying covariates were handled via list wise deletion.

The analytical sample was restricted to adults aged 15 to 85. First, we divided the sample into seven age categories (15–24, 25–34, 35–44, 45–54, 55–64, 65–74, 75–85) and seven birth cohorts (1938–1948, 1949–1958, 1959–1968, 1969–1978, 1979–1988, 1989–1998, 1999–2008), in order to identify age and cohort specific mental health trajectories. To ensure robust cohort comparisons, we then categorized respondents into four distinct birth cohorts, following Rosenberg et al. (82): Generation Z/Gen Z (born 1995 or later), Millennials (1980–1994), Generation X (1965–1979), and Baby Boomers (1945–1964). The final panel consisted of 239,244 observations for Australia and 416,453 observations for the UK. In some models, the number of observations is reduced due to the availability of covariates.

### Measures

Outcome variable: For Australia, Mental health was measured using the Mental Health Inventory (MHI-5), a subscale of the SF-36. Scores range from 0 to 100, where higher scores indicate better mental health. For the United Kingdom, Mental health was assessed using the SF-12 Mental Component Summary (MCS). We do not pool datasets, as these measures are constructed differently across datasets. The validity of these scales have been established in several previous studies (83). A detailed explanation of the Mental Health measure is given in Supplementary materials (Section 1). Empirical research supports considering these measures as conceptually comparable. Validation studies demonstrate that the MHI-5 and SF-12 MCS correlate highly with each other and with longer psychiatric screening instruments such as the General Health Questionnaire (GHQ-12) and Kessler K10 construct (84, 85). Both measures discriminate effectively between individuals with and without diagnosed mental disorders, and both are scored such that higher values indicate better mental health functioning. Nevertheless, direct comparison of regression coefficients across countries requires caution. Differences in scale construction, item content, and scoring algorithms mean that a one-unit change in MHI-5 is not metrically equivalent to a one-unit change in SF-12 MCS. This is a limitation of the present study, even though both country contexts show unique picture for mental health trajectories for different cohorts. Future studies should focus on a pooled data analysis to understand relative differences in mental health between countries. To ensure robust interpretation in each country despite these operational asymmetries, our comparative strategy relies on pattern and mechanism comparison rather than point-estimate comparison. We evaluate how the baseline structural cohort penalties for Millennials and Generation Z behave under the sequential introduction of thematic control matrices (economic precarity, family formation, and social networking) within each respective country.

For both datasets, the outcome variable was log-transformed. This transformation serves two critical statistical functions. First, population-level mental health distributions are characteristically skewed, as the majority of community-dwelling respondents cluster toward the higher (healthier) end of the scale. Log-transformation mitigates this skewness, helping to stabilize the variance and better satisfy the normality assumptions required for the residuals in our hierarchical age-period-cohort model (HAPC) sand Generalized Additive Models (GAMs). Second, employing a log-linear specification allows the estimated coefficients to be interpreted as semi-elasticities, representing the approximate percentage change in mental health associated with a one-unit change in the predictor variables.

Exposure: We tested three distinct determinants, corresponding to our hypotheses. In HAPC models, the main exposure variable is the cohort, with four categories. Along with the explanatory hypotheses, several other variables were included in the models as specified below.

1) Economic factors: Measured via equivalized household monthly income (log-transformed), employment status (employed vs. unemployed/inactive).
2) Life course social benefits: Captured through family structure (being married or in a partnered relationship, and the number of dependent children) and educational attainment (university degree vs. non-degree qualification). (Even though there are several other social conditions related to benefits, along with the hypothesis, only marital, parenthood, and education dimensions are considered in the present analysis. Future studies may look into other factors.)
3) Social Networking: To capture the social environment, we utilized the best available metrics within each panel, though we acknowledge structural differences in their operationalization. In the UK (Understanding Society) dataset, the construct captures “Perceived Social Isolation” using four subjective items: lack of companionship, feeling of being left out, feeling of isolation from others, and feeling of loneliness (Omega = 0.78). Conversely, the Australian (HILDA) construct captures “Perceived Social Support,” utilizing items that assess both functional support and subjective feelings: having someone to confide in, having someone to lean on in a problematic situation, feeling lonely, the tendency to seek help from others, and enjoying time spent with important people (Omega = 0.76). Therefore, the UK construct fundamentally indexes the subjective experience of disconnection, whereas the Australian variable leans more toward the functional availability of support. To maintain analytical transparency, we interpret these not as direct measures of objective network size, but as subjective appraisals of social integration.

Theoretical and empirical work suggests these constructs are inversely related but not perfectly substitutable. Perceived support reflects the functional quality of relationships, while perceived isolation reflects subjective appraisal of relational deficits (86). Individuals may have access to support yet still feel lonely or lack extensive networks yet do not experience isolation. Both constructs independently predict mental health outcomes, operating through partially distinct mechanisms: social support buffers against stress through tangible and emotional assistance, while loneliness affects mental health through emotional dysregulation and threat perception (87). Given this conceptual distinction, we interpret cross-national findings regarding social connectedness with appropriate caution, even though both constructs uniquely speak of quality of social networks.

*Covariates*: All models controlled for age (linear and quadratic terms to capture life-course curvature), gender, migrant status, region of residence, and baseline mental health. Baseline mental health was defined as each respondent’s first observed MHI-5/SF-12 mental health score upon entry into the analytical sample. This person-specific baseline controls for pre-existing mental health status prior to observed life course transitions, addressing potential reverse causality whereby adolescent or early adult mental health may influence both life circumstances (such as economic stability, education, etc.) and subsequent mental health trajectories.

### Analytical strategy

Firstly, to disentangle age and cohort effects, we employed restricted maximum likelihood (REML) in Generalized Additive Models (GAMs) with penalized cubic regression splines following previous studies (88). GAM is an extension of traditional linear regression, yet it uses flexible, smooth functions to capture complex, curved or non-linear patterns in data. This non-parametric approach allows for flexible, data-driven estimation of mental health trajectories over age and time without imposing rigid functional forms. We plotted predicted margins for each cohort to observe divergence in life-cycle patterns. The main equations for GAM were derived by maximizing a quadratic penalized likelihood to automatically determine smoothness, with a starting value set to 9 (10, 88). To address the serial correlation inherent in repeated measures, we incorporated a first-order autoregressive term structure for the residuals, clustered at the individual.


$$MH_{\mathrm{it}}=\alpha +\sum_{k=1}^{7}f_{k}\;(\mathrm{ag}e_{\mathrm{it}}).\overset{-}{\mathrm{ll}}\;\;(\text{Cohor}t_{i}=k)+X_{\mathrm{it}}\beta +\in_{\mathrm{it}}$$

Where $MH_{\mathrm{it}}$ is the standardized mental health score for individual i at time t. α is the global intercept. $f_{k}\;({\mathrm{age}}_{\mathrm{it}})\;$ represents the smooth function (spline) of age specifically estimated for cohort k. $\left({\text{Cohort}}_{i}=k\right)$ is an indicator function that takes the value of 1 if individual i belongs to cohort k, and 0 otherwise. $X_{\mathrm{it}}$ is a vector of parametric covariates. $\epsilon_{\mathrm{it}}$ is the normal error term. The smooth functions *f*_k_(.) were estimated using penalized cubic regression splines, with smoothing parameters selected via Restricted Maximum Likelihood (REML) to prevent overfitting. This approach allows us to formally test if the functional form of the age-mental health relationship differs by birth cohort.

Then, to understand reasons explaining generational differences, we used the sequential Hierarchical Age-Period-Cohort (HAPC) framework. Correlated random effects models are also possible to use in this context to a certain extent, though they suffer from the exact identification problem, as age, period, and cohort are perfectly collinear. To overcome these issues, we utilizes a Cross-Classified Random-Effects Model (CCREM) as suggested in the HAPC framework, a multilevel methodology proposed by Yang and Land (12, 14), which conceptualizes periods and birth cohorts as intersecting macro-level contexts rather than linear covariates. While the standard HAPC model was initially developed for repeated cross-sectional data, applying this framework to longitudinal panel data allows for significant methodological advancements. Relying on repeated cross-sections conflates true within-person aging with unobserved between-person baseline differences. By utilizing panel data, we extend the HAPC framework to explicitly model repeated measurement occasions (Level 1) nested within individuals, who are in turn cross-classified within historical periods and birth cohorts (Level 2).

The level 1 equation is specified as:


$$Y_{\mathrm{ti}(\mathrm{jc})}=\beta_{0i(\mathrm{jc})}+\beta_{1}\mathrm{Ag}e_{\mathrm{ti}(\mathrm{jc})}+\beta_{2}\mathrm{Ag}e_{\mathrm{ti}(\mathrm{jc})}^{2}+X_{\mathrm{ti}(\mathrm{jc})}\beta +e_{\mathrm{ti}(\mathrm{jc}).}$$

Where Y is the predicted mental health score at measurement occasion *t* for individual *i*, cross-classified within survey wave (period) *j* and birth cohort *c*. The biological life-course trajectory is modeled using Age and Age squared. The vector X represents a set of time-varying sociodemographic and economic mechanisms (e.g., employment status, income, marital status, and social networking), and *e* is the time-specific residual error. To account for unobserved individual heterogeneity and omitted variable bias, the between-person variation is modeled incorporating the Mundlak means for time varying covariates (income, marital status, number of children, higher education, age, social networks) (89, 90):


$$\beta_{0i(\mathrm{jc})}=\gamma_{00(\mathrm{jc})}+{\bar{x}}_{i}\gamma +Z_{i}\delta +u_{0i}$$

Here, the individual intercept *β* is a function of the grand mean γ , time-invariant individual characteristics Z (e.g., gender, immigrant status), and a person-specific random effect $u_{0i}$ , representing stable psychological traits. Crucially, we include ${\bar{x}}_{i}$ , the person-specific longitudinal means of all time-varying covariates. This specification relaxes the restrictive random-effects assumption that covariates are uncorrelated with the error term, allowing the model to simulate fixed-effects stringency while retaining the ability to estimate macro-level temporal contexts.

The Level 2 (macro-contextual) equation models the cross-classification of time and generation.


$$\begin{array}{c} \gamma_{00(\mathrm{jc})}=\pi_{0}+v_{0j}+w_{0c} \end{array}$$

Where $\pi_{0}$ is the global intercept, $v_{0j}$ is the random-effect variance representing periods (assumed to be normally distributed $\begin{array}{c} v_{0j}∼N(0,\tau_{v}) \end{array}$ , and $w_{0c}$ is the random-effect variance representing the structural cohorts $\begin{array}{c} w_{0c}∼N(0,\tau_{w}) \end{array}$ . By estimating periods and cohorts as random effects and introducing explicit social and economic mechanisms into the fixed-effects matrix, this design avoids the arbitrary mathematical constraints that often bias standard HAPC models (43). This mechanism-based identification strategy explicitly parameterizes the lived structural conditions of the respondents, allowing for a mathematically identified and theoretically grounded estimation of the remaining structural cohort variances. All models are estimated using Restricted Maximum Likelihood (REML) to ensure unbiased variance components.

We estimated six nested models to isolate the drivers of the cohort gap. Model 1 includes no socioeconomic controls other than demographics. Model 2 adds Income and Employment. Model 3 adds Marital Status, Children, and Education. Model 4 includes Social Networks/Support. Model 5 adjusts for all domains simultaneously. Model 6 restricts the sample to ages between 25 and 65. All models include demographic and regional controls. GAM models were implemented using R, and HAPC models were implemented using Stata 19.5, with standard errors clustered at the individual level to account for serial correlation within the panel.

A central methodological challenge in this longitudinal analysis is the classical Age-Period-Cohort identification problem, arising from the perfect linear dependency among the three temporal dimensions. To overcome this, we employed a two-step modeling strategy designed to systematically disentangle these effects. First, to map the life-course trajectories without imposing restrictive linear assumptions, we utilized Generalized Additive Models with penalized cubic regression splines (88). By allowing the functional form of the age-mental health relationship to curve freely, this approach breaks the strict linear collinearity inherent in standard APC models. This non-parametric specification enables us to visually and statistically isolate distinct cohort intercepts and diverging age trajectories, providing a clear baseline for generational comparison.

We acknowledge the significant methodological concern regarding non-random attrition in longitudinal designs, particularly among younger and disadvantaged cohorts over a long observational window. To mitigate the impact of attrition bias on the estimated cohort trajectories, we have mainly used the Mundlak specification, which inherently controls for time-invariant unobserved heterogeneity; by partitioning out person-specific means, the model adjusts for stable baseline characteristics associated with the propensity to attrit. Non-random attrition is addressed in longitudinal studies using various approaches, though the use of those mechanisms in APC has yet to be developed. As a result, non-random attrition is frequently acknowledged as a limitation in many APC studies that previously used longitudinal data, and this is also true in our studies.

## Results

Table 1 presents the descriptive analysis for Australia and the United Kingdom. In both contexts, distinct mental health outcomes are evident across the life course, with mental health scores increasing monotonically with age; the youngest age group (15–24) reports the lowest mean scores (Australia: 4.21; UK: 3.81), while the oldest group (75–85) reports the highest. This is starkly reflected in the cohort breakdown, where Gen Z exhibits a substantial mental health deficit (Australia: 4.16; UK: 3.76) compared to Baby Boomers (Australia: 4.28; UK: 3.90) and Generation X.

**Table 1:** Descriptive analysis.

Variables	Australia	United Kingdom
	Mean	Mean
Mental health	MHI-5 scale (log) 4.25 (0.001)	SF-12 scale (log) 3.85 (0.001)
Age groups (mean mental health)		
15–24	4.21 (0.001)	3.81 (0.001)
25–34	4.22 (0.001)	3.81 (0.001)
35–44	4.24 (0.001)	3.83 (0.001)
45–54	4.25 (0.001)	3.84 (0.001)
55–64	4.27 (0.001)	3.88 (0.001)
65–74	4.31 (0.001)	3.93 (0.001)
75–85	4.31 (0.001)	3.93 (0.001)
Cohorts (mean mental health)		
1938–1948 (older Boomers)	4.30 (0.001)	3.93 (0.001)
1949–1958 (younger Boomers)	4.28 (0.001)	3.89 (0.001)
1959–1968 (early Gen X)	4.25 (0.001)	3.85 (0.001)
1969–1978 (late Gen X)	4.25 (0.001)	3.84 (0.001)
1979–1988 (early Millennial)	4.23 (0.001)	3.81 (0.001)
1989–1998 (late Millennial)	4.20 (0.001)	3.80 (0.001)
1999–2008 (Gen Z)	4.13 (0.003)	3.74 (0.002)
Cohorts - 4 categories (mean mental health)		
1945–1964 (Boomers)	4.28 (0.001)	3.90 (0.001)
1965–1979 (Gen X)	4.25 (0.001)	3.84 (0.001)
1980–1994 (Millennial)	4.23 (0.001)	3.81 (0.001)
1995+ (Gen Z)	4.16 (0.002)	3.76 (0.001)
Age	36.66 (0.033)	48.52 (0.024)
Men	23,562 (49.63%)	47,159 (47.31%)
Women	23,916 (50.37%)	52,513 (52.68%)
Married (in a partnered relationship)	18,753 (39.5%)	56,346 (56.52%)
Not in a partnered relationship	28,725 (60.5%)	43,345 (43.48%)
Employed	19,413 (40.89%)	49,049 (49.20%)
Not employed	28,065 (59.11%)	44,893 (45.03%)
Children	0.7 (0.001) Min Max: 0–12	0.45 (0.001) Min Max: 0–10
Social networks (social support—AU/social isolation—the UK)	2.35 (0.002) Min Max: 1–7	1.47 (0.001) Min Max: 1–3
Highly educated (bachelor’s or above)	6,950 (14.64%)	25,787 (25.87%)
Less educated (Less than Bachelors)	40,520 (85.36%)	59,486 (59.67%)
Income (log)	10.36 (0.002)	7.13 (0.001)
Immigrant background/foreign born	23,466 (49.42%)	20,970 (21.03%)
Country born individuals	24,012 (50.58%)	77,289 (77.53%)

**Figure 1:**
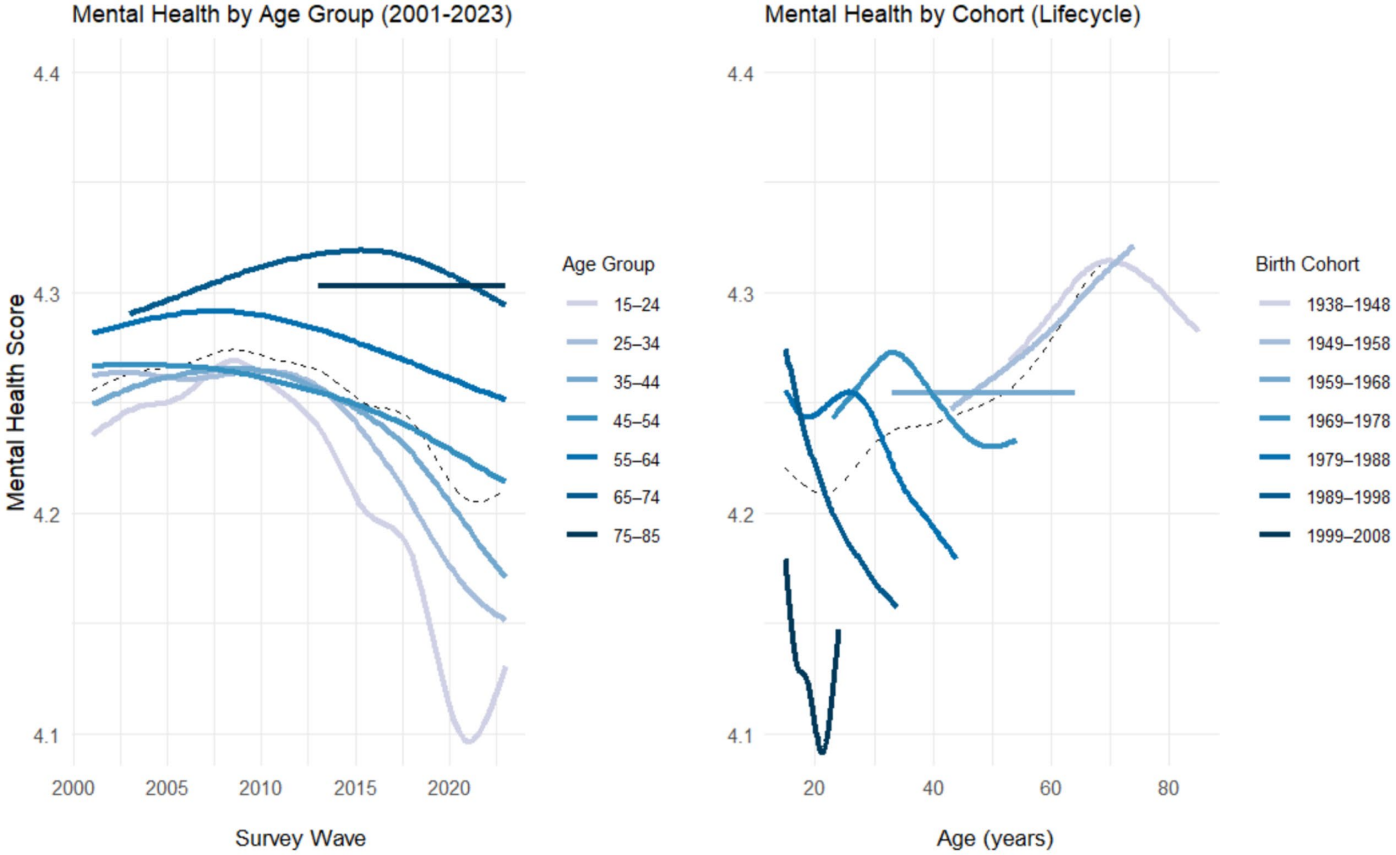
Mental Health Trajectories of Australia. This figure illustrates the trajectories of mental health for Australia across distinct age groups over survey waves (Left Panel) and birth cohorts over the life course (Right Panel). Higher values in the mental health score represent higher mental health.

The Left Panel (Period Effects) in Figure 1 reveals a sharp divergence in mental health trends in Australia. While older age groups maintain relatively stable trajectories, mental health has notably deteriorated for younger groups, specifically those aged 15–24 and 25–34, particularly following 2009. The deviation from the mean trend (dotted line, representing the average period effect) indicates that this decline has accelerated in recent waves. A notable exception occurs post-2021, when the 15–24 age group shows a slight upward recovery, although their absolute mental health levels remain significantly below those of older cohorts.

The Right Panel (Cohort Effects) in Figure 1 highlights a marked generational gradient. Recent generations, specifically Millennials and Gen Z, have experienced a precipitous decline in mental health as they age. In contrast, trend lines for older cohorts remain relatively flat; this linearity reflects the penalized smoothing spline (GAM), which determined that no additional degrees of freedom were required to support curvature, indicating stability in mental health for these groups. Crucially, the Australian Gen Z cohort shows a distinct ‘U-shaped’ trajectory: after an initial decline, it rises sharply after age 20. This suggests that while Australian youth face significant early distress, there is evidence of stabilization in early adulthood. Overall, individuals born after 1979 exhibit a significantly higher rate of mental health decline compared to pre-1980 cohorts, implying substantial cohort heterogeneity.

**Figure 2:**
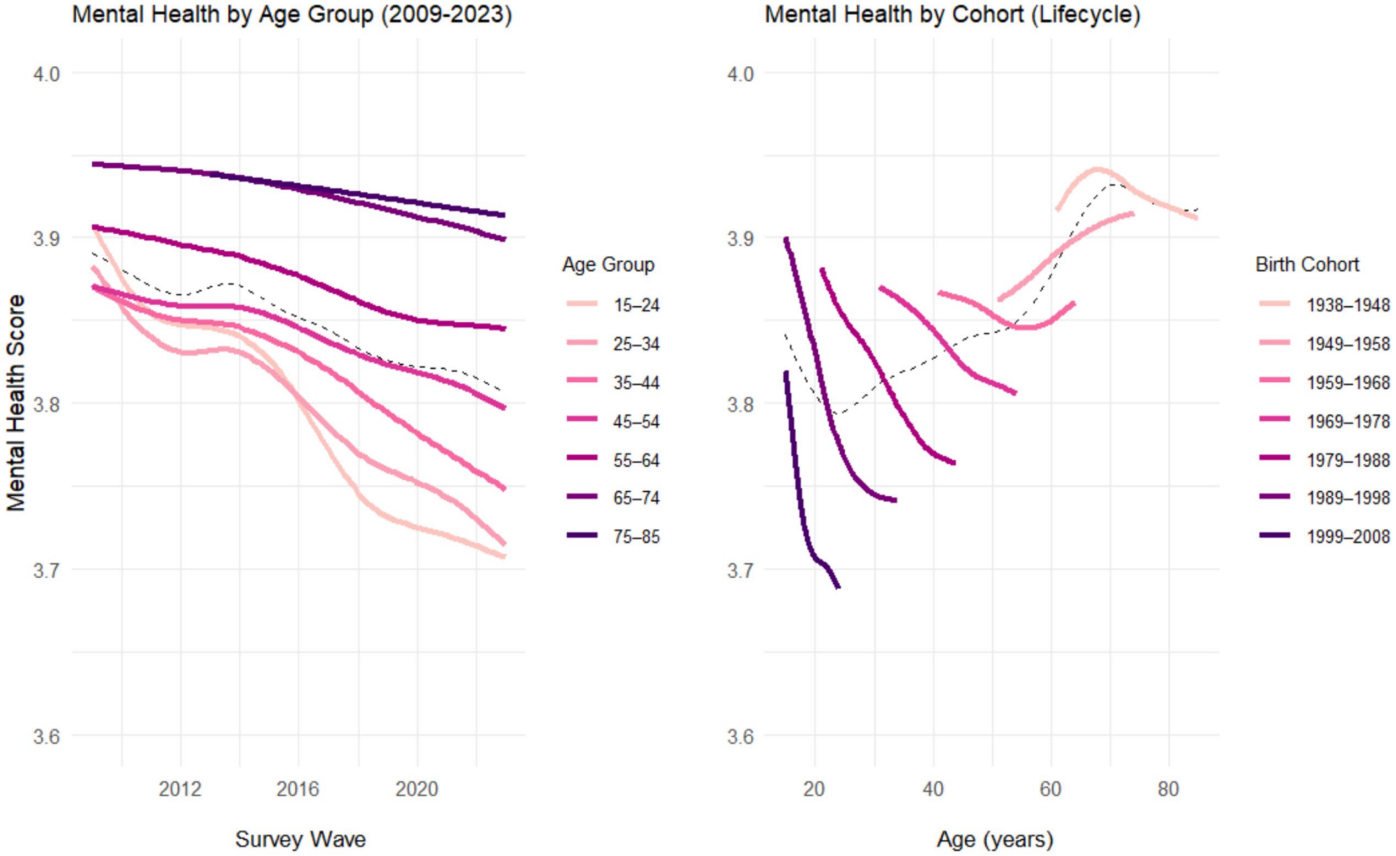
Mental Health Trajectories of the UK. This figure illustrates the trajectories of mental health for the UK across distinct age groups over survey waves (Left Panel) and birth cohorts over the life course (Right Panel). Higher values in the mental health score represent higher mental health.

Figure 2 presents the analysis for the United Kingdom, revealing trends that are directionally comparable to Australia but significantly more severe in magnitude. The Left Panel demonstrates that the deterioration of mental health among younger age groups (15–24 and 25–34) is profound. The deviation from the global trend line is far wider than in the Australian context, signaling a deeper structural decline. Furthermore, the “age of divergence” is higher in the UK: while mental health declines typically onset below age 45 in Australia, in the UK, this negative trajectory extends to individuals up to age 55. The Right Panel confirms that British Gen Z and Millennials are profoundly disadvantaged. Unlike their Australian counterparts, UK Gen Z shows no evidence of recovery; their trajectory continues to decline dramatically with age. Millennials display a minor inflection point after age 33, yet their trajectory remains far below the global trend, indicating no substantive recovery over the life course. This contrast is pivotal: while Australian youth show signs of resilience (potentially linked to the ‘autonomy’ mechanism identified in our models), UK youth appear trapped in a persistent downward spiral, consistent with the social networking hypothesis.

**Figure 3:**
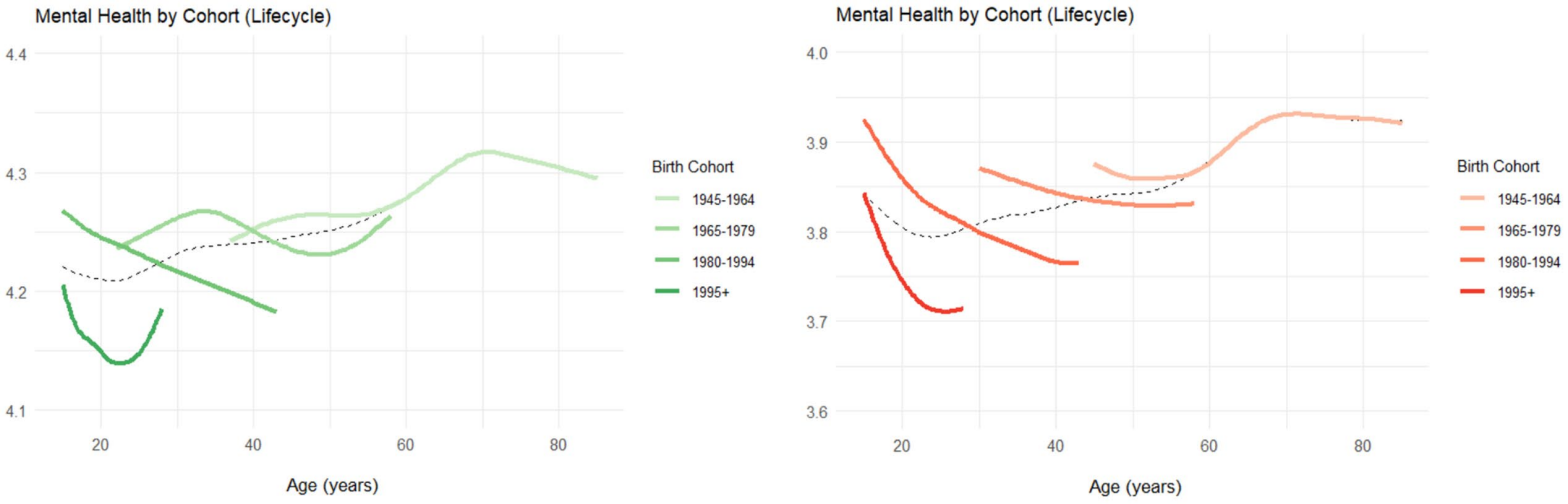
Four birth-cohort differences in mental health. Note: Generational mental health differences. Left Panel (Australia), Right Panel (the UK).

To facilitate robust comparative analysis, we categorized respondents into four distinct birth cohorts: Gen Z (1995+), Millennials (1980–1994), Gen X (1965–1979), and Baby Boomers (1945–1964). Figure 3 reveals a robust generational gradient in both countries. Consistently, younger cohorts (Millennials and Gen Z) exhibit significant mental health disadvantages compared to older generations (Gen X and Baby Boomers). However, a divergence in trajectory shape is evident. In the UK (Right Panel), the disadvantage for younger cohorts appears persistent and linear, suggesting a continuous accumulation of distress. In Australia (Left Panel), while Gen Z begins with a severe deficit, they exhibit a distinct “U-shaped” recovery pattern in early adulthood. These findings confirm the existence of a “cohort penalty” among youth, motivating a subsequent multivariate analysis to determine whether this gap is driven by observable social and economic factors.

Across the six models presented in Table 2, the variance attributed to birth cohort consistently surpasses the variance attributed to periods, indicating that the mental health difference is primarily a generational phenomenon. In Model 1, the standard deviation for the cohort random effect (0.0330) is more than thrice the period effect (0.0090). Models 2 through 5 introduce economic and social controls, yet the period variance remains small and relatively flat. Meanwhile, the cohort variance experiences minor fluctuations, demonstrating a persistent generational mental health difference that remains the dominant macro-level driver even after controlling for individual-level economic and social factors.

**Table 2:** Mental health trajectories in Australia.

Mental Health	Model 1	Model 2	Model 3	Model 4	Model 5	Model 6
Variance attributed to birth cohorts						
Baby Boomers (1945–1964)	0.031 (0.017) [ci: −0.002 to 0.065]	0.031 (0.016) [ci: −0.001 to 0.064]	0.038 (0.020) [ci: −0.001 to 0.007]	0.035 (0.018) [ci: 0.001–0.069]	0.035 (0.017) [ci: 0.001–0.069]	0.018 (0.011) [ci: −0.003 to 0.039]
Gen X (1965–1979)	0.015 (0.017) [ci: −0.17 to 0.048]	0.013 (0.016) [ci: −0.018 to 0.045]	0.018 (0.020) [ci: −0.020 to 0.056]	0.015 (0.017) [ci: −0.018 to 0.049]	0.014 (0.017) [ci: −0.19 to 0.046]	0.012 (0.010) [ci: −0.030 to 0.009]
Millennials (1980–1994)	−0.002 (0.016) [ci: −0.034 to 0.031]	−0.002 (0.016) [ci: −0.034 to 0.029]	−0.004 (0.020) [ci: −0.043 to 0.034]	−0.007 (0.017) [ci: −0.040 to 0.027]	−0.006 (0.016) [ci: −0.039 to 0.026]	−0.010 (0.010) [ci: −0.030 to 0.009]
Gen Z (1995+)	−0.045 (0.017) [ci: −0.079 to (−0.009)]	−0.042 (0.017) [ci: −0.075 to (−0.008)]	−0.051 (0.020) [ci: −0.091 to (−0.011)]	−0.044 (0.018) [ci: −0.079 to (−0.009)]	−0.042 (0.018) [ci: −0.077 to (−0.007)]	−0.019 (0.012) [ci: −0.045 to 0.005]
Fixed components						
Female	−0.011^***^	0.0003	−0.010^***^	−0.035^***^	−0.028^***^	−0.026^***^
	(0.002)	(0.002)	(0.002)	(0.002)	(0.002)	(0.003)
Age	−0.004^***^	−0.005^***^	−0.004^***^	−0.002^***^	−0.003^***^	−0.008^***^
	(0.0001)	(0.0001)	(0.0001)	(0.0001)	(0.001)	(0.0001)
Age squared	0.0001^***^	0.0001^***^	0.0001^***^	0.0001^**^	0.0001^***^	0.0001^***^
	(0.0001)	(0.0001)	(0.0001)	(0.0001)	(0.0001)	(0.0001)
Log income		−0.001			−0.001^*^	−0.001
		(0.001)			(0.001)	(0.001)
Employed		0.031^***^			0.028^***^	0.041^***^
		(0.002)			(0.002)	(0.002)
Married			0.038^***^		0.024^***^	0.027^***^
			(0.002)		(0.001)	(0.002)
Social support				0.080^***^	0.078^***^	0.085^***^
				(0.001)	(0.001)	(0.001)
Children			−0.009^***^		−0.003^***^	−0.001
			(0.001)		(0.001)	(0.001)
Higher education			0.018^***^		0.001	0.001
			(0.002)		(0.002)	(0.002)
Intercept	1.691^***^	1.710^***^	1.707^***^	2.309^***^	2.345^***^	2.601^***^
	(0.026)	(0.030)	(0.023)	(0.026)	(0.028)	(0.036)
Standard deviations of the random effects/variance components						
Cohort	0.0330	0.0318	0.0388	0.0341	0.0335	0.0192
	(0.0144)	(0.0139)	(0.0166)	(0.0146)	(0.0144)	(0.0107)
Period	0.0090	0.0097	0.0088	0.0070	0.0074	0.0102
	(0.0014)	(0.0017)	(0.0014)	(0.0012)	(0.0014)	(0.0021)
Individual	0.1532	0.1500	0.1525	0.1347	0.1332	0.1404
	(0.0009)	(0.0009)	(0.0009)	(0.0008)	(0.0008)	(0.0010)
Residual	0.2088	0.2086	0.2085	0.2016	0.2010	0.2043
	(0.0003)	(0.0003)	(0.0003)	(0.0003)	(0.0003)	(0.0004)
Num. Obs.	239,244	239,244	239,244	239,244	239,244	175,514
AIC	−32,241.78	−33,232.85	−32,853.48	−54,209.81	−53,751.48	−31,365.02
BIC	−32,106.77	−33,056.3	−32,666.55	−54,053.54	−53,502.24	−31,123.21

Standard errors clustered at the individual level and are given in parentheses. All models include Mundlak mean for time-varying covariates, immigrant state, baseline mental health, and place of residence (region). In Model 6, age is restricted to 25–65. Variance attributed to birth cohorts are generated from variance components in mixed effects models. The comparison of cohorts should be done according to Table 3, where between cohort differences are presented. Confidence intervals in variance attributed to birth cohosts represent in accordance with the grand mean mental health score. Therefore, P vale significance is not given for cohort differences in this table. Generational difference significance should be thus obtained from Table 3. Except for female and higher education (between variation), all other covariates represent within variation.

^+^*p* < 0.1; ^*^p < 0.05, ^**^p < 0.01, ^***^p < 0.001.

The Empirical Bayes (EB) estimates extracted from the random components in Table 2 show a persistent decline in mental health, mainly among Gen Z. While examining the coefficients against the grand mean shows Gen Z as the only cohort with a statistically significant absolute penalty, post-estimation contrast tests (Table 3) reveal a profound relative gap between the youngest and oldest generations. In the fully specified unrestricted model (Model 5), the mental health of Gen Z is significantly lower than both Baby Boomers (Difference = 0.077, SE = 0.025, *p* < 0.01) and Gen X (Difference = 0.056, SE = 0.025, *p* < 0.05). Millennials, representing a transitional baseline, also exhibit a marginally significant deficit when compared directly to the Baby Boomer premium (Difference = 0.041, SE = 0.023, *p* = 0.078).

**Table 3:** Post-estimation pairwise comparisons of structural cohort differences (Australia).

Model specification	Baby Boomers vs. Gen Z difference (SE) [*p*-value]	Generation X vs. Gen Z difference (SE) [*p*-value]	Baby Boomers vs. millennials difference (SE) [*p*-value]
Model 1	0.076 (0.024)^***^ p = 0.001	0.060 (0.024)^*^ p = 0.012	0.033 (0.023) *p* = 0.158
Model 2	0.073 (0.023)^**^ p = 0.002	0.055 (0.023)^*^ p = 0.018	0.033 (0.023) *p* = 0.144
Model 3	0.089 (0.028)^**^ p = 0.002	0.069 (0.028)^*^ p = 0.014	0.042 (0.028) *p* = 0.138
Model 4	0.079 (0.026)^**^ p = 0.002	0.059 (0.025)^*^ p = 0.017	0.042 (0.025)^+^ *p* = 0.089
Model 5	0.077 (0.025)^**^ p = 0.002	0.056 (0.025)^*^ p = 0.023	0.041 (0.023)^+^ *p* = 0.080
Model 6	0.037 (0.016)^*^ p = 0.023	0.031 (0.016)^*^ p = 0.047	0.028 (0.015)^+^ *p* = 0.058

Difference scores were calculated via post-estimation contrasts of Empirical Bayes (EB) estimates from Variance attributed to birth cohorts. Standard errors of the difference are reported in parentheses.

^+^*p* < 0.1, ^*^p < 0.05, ^**^p < 0.01, ^***^p < 0.001.

**Figure 4:**
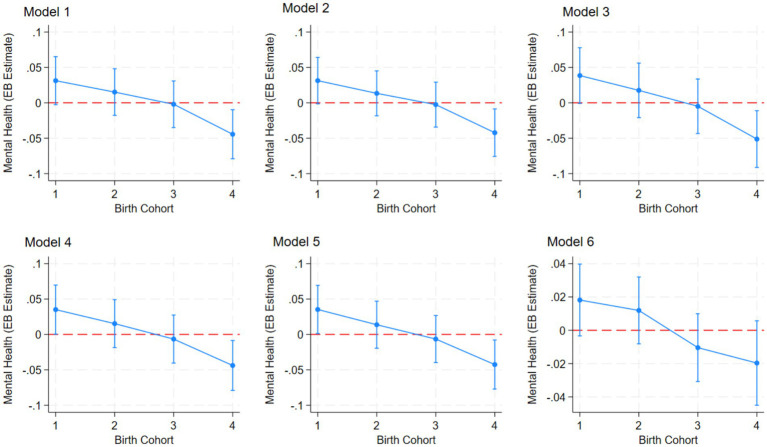
Cohort differences in predicted mental health among Australians. This figure illustrates predicted variance attributed to birth cohorts in Australia, along with the models given in Table 2. 1. Baby Boomers, 2. Gen X, 3. Millennials, 4. Gen Z.

Concerning the conflation of cohort effects with the volatility of the youth transition to adulthood, Model 6 restricts the analytical sample to established working-age adults (ages 25–65). In this model, the EB estimates for all cohorts attenuate toward the grand mean, indicating that among adults, the absolute youth mental health penalty normalizes to a certain extent. However, direct contrast tests between the cohorts prove that the relative generational differences persist even after removing the under-25 age population. Post-estimation analyses on the restricted sample show that Gen Z’s mental health remains lower than that of Baby Boomers (Difference = 0.037, SE = 0.017, *p* = 0.026). Furthermore, the gaps between Millennials and Boomers (Difference = 0.028, SE = 0.015, *p* = 0.056), as well as Gen Z and Gen X (Difference = 0.031, SE = 0.016, *p* = 0.067), remain marginally significant. Figure 4 illustrates variance attributed to random effect components of cohorts across all six models for Australia.

In the Australian context, the sequential models demonstrate that Gen Z experience a pronounced mental health deficit compared to older cohorts. In the baseline specification (Model 1), Gen Z shows a marked mental health deficit relative to older cohorts. Crucially, as the models introduce indicators for economic precarity (Model 2), family structure (Model 3), and social support (Model 4), this Gen Z penalty remains stable. However, coefficients were reduced to a certain extent in Model 2, supporting H1. In Model 3, we observe a suppression association, and hence, H2 cannot be substantiated. However, Model 4 provides an important picture, where social support is included, the coefficients are reduced significantly for Gen Z, supporting H3. However, even in the fully specified model (Model 5), Gen Z remains significantly worse off than both Baby Boomers and Gen X. Australian Millennials, by contrast, display only marginal, non-significant differences from older cohorts across all specifications. This trajectory indicates that Australia’s economic and social institutions successfully buffered Millennials during the late 2000s but are currently failing to mitigate a deeper, unobserved structural crisis that uniquely afflicts Gen Z regardless of their individual socioeconomic standing.

The attenuation of Gen Z’s mental health penalty in the adulthood sample (Model 6) suggests that the widely observed ‘generational crisis’ is more clearly understood as an acute *transition* crisis. Importantly, the severe economic precarity and disrupted social networking that define the modern school-to-work transition begin to resolve as young adults cross age 25. As individuals age into the primary labor market, whereby they secure standard employment, stabilize income, and accumulate durable social capital and family ties, they successfully rebuild their institutional and relational resources to improve mental health.

To assess the relative importance of the temporal dimensions, Variance Partitioning Coefficients (VPC) were calculated from the random-effect variance components of Model 5. Generational affiliation accounts for 1.89% of the total variance in mental health, exerting an impact more than twenty times larger than of historical periods (0.09%). The near-zero period variance indicates that the time-varying socioeconomic mechanisms included in the model successfully captured the structural historical shocks occurring across the observational window.^1^

Table 4 shows the results of HAPC models in the UK sample. At the macro-contextual level, the baseline model indicates that birth cohort has a stronger structural influence (0.0230) than period effects (0.0149). However, the sequential implementation of economic and social mechanisms reduces macro-level variations.

**Table 4:** Mental health trajectories in the UK.

Mental health	Model 1	Model 2	Model 3	Model 4	Model 5	Model 6
Variance attributed to birth cohorts						
Baby Boomers (1945–1964)	0.025 (0.011) [ci: 0.002–0.049]	0.026 (0.011) [ci: 0.003–0.047]	0.027 (0.013) [ci: 0.003–0.054]	0.023 (0.010) [ci: 0.004–0.041]	0.018 (0.008) [ci: 0.002–0.034]	−0.001 (0.001) [ci: −0.004 to 0.003]
Gen X (1965–1979)	0.011 (0.011) [ci: −0.012 to 0.034]	0.008 (0.011) [ci: −0.013-0.030]	0.012 (0.013) [ci: −0.013 to 0.036]	0.002 (0.010) [ci: −0.015 to 0.019]	−0.003 (0.007) [−0.017 to 0.011]	0.001 (0.002) [ci: −0.002 to 0.004]
Millennials (1980–1994)	−0.010 (0.012) [ci: −0.032 to (−0.013)]	−0.010 (0.010) [ci: −0.032 to 0.011]	−0.013 (0.012) [ci: −0.038 to 0.011]	−0.016 (0.010) [ci: −0.033 to 0.001]	−0.012 (0.007) [−0.026 to 0.002]	0.0003 (0.002) [ci: −0.003 to 0.003]
Gen Z (1995+)	−0.027 (0.012) [ci: −0.050 to (−0.003)]	−0.023 (0.011) [ci: −0.045 to (−0.001)]	−0.027 (0.013) [ci: −0.053 to (−0.002)]	−0.010 (0.010) [ci: −0.028 to 0.010]	−0.003 (0.008) [−0.019 to 0.013]	−0.0003 (0.002) [ci: −0.004 to 0.003]
Fixed components					
Female	−0.022^***^	−0.020^***^	−0.021^***^	−0.016^***^	−0.019^***^	−0.017^***^
	(0.002)	(0.001)	(0.001)	(0.002)	(0.002)	(0.002)
Age	−0.013^***^	−0.013^***^	−0.014^***^	−0.011^***^	−0.010^***^	−0.012^***^
	(0.001)	(0.0001)	(0.0001)	(0.0001)	(0.0001)	(0.001)
Age squared	0.0001^***^	0.0001^***^	0.0001^***^	0.0001^***^	0.0001^***^	0.0001^***^
	(0.0001)	(0.0001)	(0.0001)	(0.0001)	(0.0001)	(0.0001)
Log income		−0.002^***^			−0.001^+^	−0.002^*^
		(0.001)			(0.001)	(0.001)
Employed		0.022^***^			0.021^***^	0.028^***^
		(0.001)			(0.002)	(0.003)
Married			−0.030^*^		−0.007^+^	−0.007^*^
			(0.002)		(0.003)	(0.004)
Social isolation				−0.163^***^	−0.161^***^	−0.161^***^
				(0.001)	(0.002)	(0.002)
Children			−0.005^***^		−0.002	−0.001
			(0.001)		(0.002)	(0.001)
Higher education			0.003^**^		0.005^**^	0.008^***^
			(0.001)		(0.002)	(0.002)
Intercept	1.505^***^	1.612^***^	1.547^***^	2.707^***^	2.785^***^	2.935^***^
	(0.022)	(0.018)	(0.018)	(0.021)	(0.022)	(0.029)
Standard deviations of the random effects						
Cohort	0.0230	0.0217	0.0252	0.0172	0.0136	0.0020
	(0.0098)	(0.0093)	(0.0107)	(0.0077)	(0.0064)	(0.0040)
Wave	0.0149	0.0167	0.0153	0.0031	0.0032	0.0061
	(0.0029)	(0.0033)	(0.0030)	(0.0010)	(0.0011)	(0.0020)
Individual	0.1215	0.1226	0.1211	0.1312	0.1322	0.1338
	(0.0005)	(0.0005)	(0.0005)	(0.0007)	(0.0008)	(0.0010)
Residual	0.1913	0.1892	0.1913	0.1847	0.1839	0.1869
	(0.0002)	(0.0002)	(0.0002)	(0.0004)	(0.0004)	(0.0005)
Num. Obs.	416,453	416,453	416,453	179,859	149,094	96,068
AIC	−20,141.91	−33,232.85	−32,853.48	−51,406.01	−42,272.58	−23,779.34
BIC	−20,013.05	−33,056.3	−32,666.55	−51,254.51	−42,034.68	−23,551.99

Standard errors clustered at the individual level and are given in parentheses. All models include Mundlak mean for time-varying covariates, immigrant state, baseline mental health, and place of residence (region). In Model 6, age is restricted to 25–65. Variance attributed to birth cohorts are generated from variance components in mixed effects models. The comparison of cohorts should be done according to Table 5, where between cohort differences are presented. Confidence intervals in variance attributed to birth cohosts represent in accordance with the grand mean mental health score. Therefore, P vale significance is not given for cohort differences in this table. Generational difference significance should be thus obtained from Table 5. Except for female and higher education (between variation), all other covariates represent within variation.

^+^*p* < 0.1, ^*^p < 0.05, ^**^p < 0.01, ^***^p < 0.001.

Table 4 demonstrates that Gen Z’s mental health deficit is sensitive to structural socioeconomic mediators. In Model 1, Gen Z shows a significant mental health penalty relative to both Baby Boomers (*p* < 0.001) and Gen X (*p* = 0.020). However, when perceived isolation control (vitality of social connectedness) is introduced in Model 4, the gap between Gen Z and Gen X disappears. By the fully specified Model 5, Gen X and Gen Z share the exact same structural coefficient (−0.003). This indicates that the Gen Z crisis relative to the middle-aged baseline is not an unobserved cultural scar; it is entirely mediated by the contemporary decline in social connectedness and economic agency. Generational differences are given in Table 5 for the UK.

**Table 5:** Post-estimation pairwise comparisons of structural cohort differences (the UK).

Model specification	Baby Boomers vs. Millennials difference (SE) [*p*-value]	Baby Boomers vs. Gen Z difference (SE) [*p*-value]	Generation X vs. Gen Z difference (SE) [*p*-value]
Model 1	0.035 (0.016)^*^ p = 0.032	0.052 (0.016)^***^ p = 0.001	0.038 (0.016)^*^ p = 0.020
Model 2	0.036 (0.015)^*^ p = 0.015	0.049 (0.016)^**^ p = 0.002	0.031 (0.016)^*^ p = 0.046
Model 3	0.040 (0.018)^*^ p = 0.024	0.054 (0.018)^**^ p = 0.003	0.039 (0.018)^*^ p = 0.034
Model 4	0.039 (0.014)^**^ p = 0.006	0.033 (0.014)^*^ p = 0.020	0.012 (0.014) *p* = 0.396
Model 5	0.030 (0.011)^**^ p = 0.005	0.021 (0.011) ^+^ *p* = 0.064	0.000 (0.011) *p* = 1.000
Model 6	0.001 (0.002) *p* = 0.561	0.001 (0.002) *p* = 0.754	0.001 (0.003) *p* = 0.646

Difference scores were calculated via post-estimation contrasts of Empirical Bayes (EB) estimates from Variance attributed to birth cohorts. Standard errors of the difference are reported in parentheses.

^+^*p* < 0.1, ^*^p < 0.05, ^**^p < 0.01, ^***^p < 0.001.

**Figure 5:**
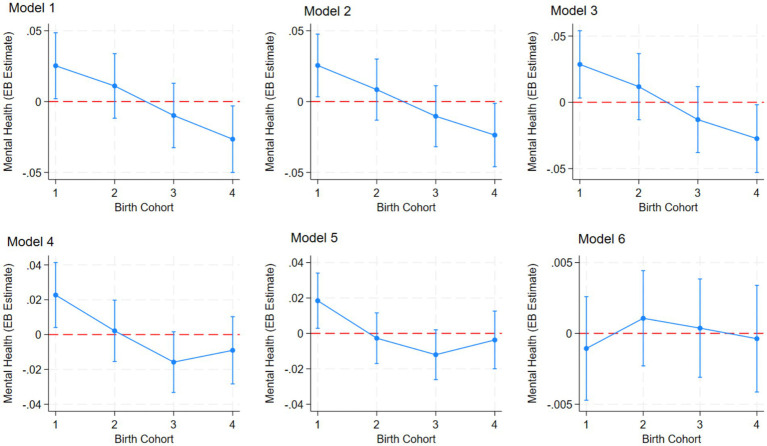
Cohort differences in predicted mental health in the UK. This figure illustrates predicted variance attributed to birth cohorts in the UK, along with the models given in Table 4. 1. Baby Boomers, 2. Gen X, 3. Millennials, 4. Gen Z.

Contrasting with Gen Z, the UK Millennials retain a clear mental health decline. Even in the fully controlled specification (Model 5), the gap between Baby Boomers and Millennials remains highly significant (0.030, *p* = 0.005), while the gap between Boomers and Gen Z drops to marginal significance (*p* = 0.064). This suggests that in the UK, Millennials (who came of age during the prolonged fallout of the 2008 financial crisis and subsequent austerity measures) bear a deeper structural ‘scar’ that cannot be fully explained away by current, contemporaneous measures of economic or social networks. Variance attributed to birth cohorts of the UK is depicted in Figure 5.

The analysis for the UK presents an inverted generational dynamic, where the inclusion of mechanisms fully explains Gen Z’s distress while exposing a deeper Millennial scar. Initially, UK Gen Z demonstrates a significant baseline penalty relative to older cohorts (Model 1). However, unlike in Australia, this deficit is sensitive to structural mediators. The introduction of economic precarity controls (Model 2) and, most notably, perceived social isolation (Model 4), heavily attenuates this gap, reducing the Gen Z penalty to marginal non-significance in the fully controlled model (Model 5). This proves that the UK’s Gen Z crisis is almost entirely driven by an immediate, contemporaneous lack of economic agency and social connectedness, supporting H1 and H3. However, H2 cannot be supported even in the UK, yet according to Model 5, conditions proposed in H2 tend to work in tandem with other economic and social connectivity processes.

Despite the varying vulnerabilities of Millennials and Gen Z, the age-restricted sensitivity analysis (Model 6) provides a conclusion about the nature of the generational divide. When restricting the UK sample to adults (ages 25–65), every single pairwise contrast collapses to near-zero. The standard errors shrink, and all statistical significance disappears. This confirms that the younger cohorts are bound to the severe structural penalties in mental health observed in the unrestricted models. The macro-level variance is predominantly concentrated at the generational level: birth cohort accounts for 0.36% of the total variance in mental health, exerting larger than that of contemporaneous historical periods (0.02%).

## Discussion

While the physical health of the United Kingdom and Australian populations has stabilized to a greater extent over the past two decades, mental health has followed a divergent and downward trajectory (33, 36). Our analysis highlights a stark decline in mental health, particularly among younger generations, with important generational heterogeneity. This deterioration is not uniformly distributed but is heavily stratified by generation. By disentangling Age, Period, and Cohort effects, our study rejects the narrative that youth distress is merely a function of transient life-cycle stages. In both countries the variance attributed to birth cohorts substantially exceeds that of periods, indicating that the decline is primarily a cohort phenomenon. The mechanisms underlying the cohort penalty differ sharply between the two countries. In the UK, the Gen Z deficit is almost mediated by perceived social isolation and, to a lesser degree, economic agency: once these are controlled, Gen Z is statistically indistinguishable from older cohorts. In Australia, the Gen Z penalty survives all the models and is only substantially reduced by occupational autonomy, with approximately 55% of the deficit remaining unexplained.

If distress were driven solely by the developmental transition to adulthood, the mental health baselines for Gen Z and Millennials would mirror those of previous generations at the same life stage. Instead, our trajectory analysis reveals distinct cohort intercepts: Gen Z enters adulthood with a significantly deeper mental health deficit than Millennials or Gen X did, confirming the existence of a lasting vulnerability imprinted by specific socio-historical conditions. While Millennials exhibit a chronic disadvantage accumulated over time, Gen Z shows an acute deficit, starting from a historically low baseline. Crucially, our findings highlight a cross-national difference; unlike the linear decline observed in the UK, Australian Gen Z exhibits a recovery pattern after age 20, though their absolute levels of mental health remain significantly below those of older cohorts. This corrects several previous findings that Gen Z would further loose mental health (10, 35), indicating that they have started to recover.

In the United Kingdom, the drivers of this decline appear to be distinctly stratified by cohort, reflecting a duality of social and economic exclusion. Our findings reveal that the mental health decline for British Gen Z is largely mediated by the vitality of social networking. The mental health deficit vanishes completely when controlling for social networks, implying that the crisis is not intrinsic to the cohort but is a symptom of social fragmentation (71). Despite being the most digitally connected generation (73), British youth suffer from a profound deficit in the tangible community support structures, including companionship, neighborhood cohesion, and face-to-face interaction, that buffered previous generations. The lack of recovery in the UK trajectories suggests that without social repair, this cohort effect threatens to become a permanent health inequality. Because the UK measure indexes subjective isolation rather than objective network structure, and because loneliness is conceptually proximate to psychological distress, part of this mediation may reflect shared method variance. The finding is therefore best read as evidence that the British Gen Z crisis is constituted by the experience of disconnection, while remaining agnostic about whether objective network erosion or appraisal processes are the proximate cause.

The British Millennials’ pattern is different. Contrary to the expectation that contemporaneous socioeconomic and relational controls would absorb their disadvantage, the Boomer to Millennial gap remains highly significant in the fully specified model (0.030, *p* = 0.005). British Millennials, who entered the labor market during the prolonged fallout of the 2008 financial crisis and a decade of austerity, carry a residual penalty that current income, employment, family status, and social connectedness cannot explain. This is the signature of genuine cohort scarring: a disadvantage acquired during formative years that persists net of present circumstances. The UK case thus combines two distinct pathologies, a mediated crisis of connection among Gen Z and an unmediated historical scar among Millennials.

The Australian context presents a more resistant picture. Unlike the UK, where standard models successfully resolved the generational gaps, Australian Millennials and Gen Z retain significant residual penalties even after accounting for socioeconomic mediators. The U-shaped recovery of Australian Gen Z after age 20 is a substantive contribution. Previous Australian work projected continued deterioration for this cohort, though our trajectory analysis indicates instead that stabilization begins in early adulthood, even though absolute levels remain well below those of older cohorts. One plausible interpretation, consistent with the autonomy mechanism, is that entry into the primary labor market restores a degree of agency that the school-to-work transition denies (91).

In the UK, the mental health decline for Gen Z is largely mediated by their social environment. However, given the subjective nature of our UK metrics, this is more accurately diagnosed as a crisis of perceived social disconnection rather than a purely structural deficit in network size. The ‘Gen Z deficit’ vanishes when controlling for internalized feelings of isolation, loneliness, and a lack of companionship. Because subjective feelings of loneliness are conceptually proximate to symptoms of psychological distress, this strong statistical mediation is somewhat expected, yet it shows a profound, internalized deficit in community belonging among British youth. In contrast, the mental health penalty among Australian Gen Z persists even after controlling for their social environment. Although the Australian metric captures both subjective loneliness and the functional presence of support, such as having someone to confide in or lean on, accounting for these factors does not resolve their generational deficit. This divergence reveals a fundamental difference in etiology: while British youth are undeniably experiencing a historically unprecedented level of subjective isolation, the distress of Australian youth operates largely independently of their perceived social support. This comparative finding reinforces our conclusion that the Australian decline is not primarily a crisis of connection, but rather a structural crisis rooted in restricted agency and broader macro-environmental stressors.

Our findings extend the literature on youth mental health by identifying the mechanism of decline rather than just its prevalence (2, 8, 75). Botha and Burns (10, 35) previously identified that younger Australians were experiencing a mental health depreciation; our study advances this by rejecting the hypothesis that this is solely a socioeconomic problem, instead highlighting the roles of agency. Globally, these trends align with reports from the National Institute of Mental Health (USA) and the OECD (92), which project that by 2025, nearly half of the population in developed economies may experience mental ill-health, driven by contemporary unrest, labor market competition, and social disconnection. The UK’s profile (characterized by widening inequality and ethnicity-based disparities) mirrors challenges seen in other migrant-hosting nations like Germany and the USA (2, 5, 93), where racialized minorities and second-generation immigrants face compounded mental health risks. By controlling for immigrant status in our models, we isolate the generational signal from the migration effect, though future research should explicitly examine the intersection of migration background and cohort scarring.

To ensure the validity of our conclusions, we conducted certain sensitivity analyses. We confirmed that while women are generally more disadvantaged in mental health outcomes than men, the trajectory of generational decline is comparable across men and women in both countries (Supplementary Figures 5–8). Re-estimating our models using the Kessler Psychological Distress Scale brought robust results, confirming that the cohort penalty is not an artifact of the specific instrument used (Supplementary Figure 9). For both countries, in addition to GAM models, socioeconomic variable-adjusted trajectory plots were obtained (Supplementary Figures 1–4), which are comparable to our GAM results. Therefore, studies are recommended to uncover residual mental health deficit observed among Australian Gen Z, after adjusting for structural conditions.

Moreover, certain limitations warrant caution. First, the design is observational; despite the Mundlak specification, unmeasured time-varying confounders cannot be excluded, and the mediation estimates are associational rather than causal. Second, the outcome and social network measures differ between datasets. The MHI-5 and SF-12 MCS are highly correlated and conceptually comparable (84, 85), but coefficients are not metrically equivalent, and our comparative claims accordingly rest on within-country patterns of attenuation rather than cross-country point estimates. The UK social measure indexes perceived isolation, while the Australian measure leans toward functional support; these constructs are related but not substitutable (86). Third, the age-restricted UK model (Model 6) should be interpreted carefully: because the oldest Gen Z respondents were 28 in 2023, the 25 to 65 restriction retains only a small Gen Z subsample, and the collapse of all contrasts in that model partly reflects reduced power rather than the resolution of generational differences. A critical theoretical implication of our findings is that the parameterized economic and social mechanisms do not operate uniformly across the life course; rather, their psychological utility is heavily contingent upon the respondent’s age group. Within the younger cohorts, standard institutional anchors often function atypically due to shifting macroeconomic realities. Fourth, non-random attrition and selective survivorship likely inflate the apparent advantage of older cohorts; the true generational penalty may be somewhat smaller than unadjusted estimates suggest, although the acute Gen Z deficits at the onset of adulthood are too large to be artifacts of survivorship alone.

The divergence in drivers between the two countries necessitates a targeted policy response rather than a uniform mental health strategy. In the United Kingdom, where youth distress is driven by social fragmentation, expanding clinical services alone is insufficient; policy must pivot toward s*ocial prescribing* and the restoration of community infrastructure to rebuild the social networking that younger cohorts lack. Conversely, in Australia, where distress persists despite socioeconomic controls, the solution lies in structural reform. Addressing this requires labor market regulations that enhance occupational autonomy for young workers, alongside public health strategies that explicitly validate and manage macro-environmental stressors, such as climate anxiety, which disproportionately burden the youngest generation.

## Data Availability

This paper uses unit record data from Household, Income and Labour Dynamics in Australia Survey [HILDA] conducted by the Australian Government Department of Social Services (DSS) and data from Understanding Society: Innovation Panel, Waves 1-17, 2008-2024 (94). Replication materials can be found here: https://osf.io/aeypm. Further inquiries can be directed to the corresponding author.

## Footnotes

1 Consistent with established longitudinal research on subjective well-being, the largest portion of variance (29.91%) remains at the individual level, indicating the necessity of the CRE specification to isolate within-person trajectories from unobserved baseline heterogeneity.
