# Supplementary material for: Generational differences in mental health and drivers of youth mental health decline in Australia and the United Kingdom
Source: Front Public Health. 2026 Jul 14;14:1844747. doi: 10.3389/fpubh.2026.1844747 (PMC13407260; doi:10.3389/fpubh.2026.1844747)

**Generational difference in mental health and drivers of youth mental health decline in Australia and the United Kingdom**

**Supplementary Materials**

**Section 1. Scale descriptions**

**MHI 5 Scales**

The MHI-5 is a subscale derived from the SF-36 health survey, comprising five items that assess both positive and negative dimensions of mental health. This well-validated scale has been widely used in population mental health research. Respondents indicate how often they experienced each of the following during the past four weeks: (1) been a nervous person, (2) felt so down in the dumps that nothing could cheer you up, (3) felt calm and peaceful, (4) felt down, and (5) been a happy person. Responses are recorded on a six-point scale ranging from “all of the time” to “none of the time”. Following Ware et al. (2000), each item was scored from 0 to 5 and recoded so that higher scores represent better mental health. The scale demonstrates strong psychometric properties, with internal consistency comparable to longer instruments such as the Kessler Psychological Distress Scale (K10).

**SF-12 Mental Component Summary (MCS).**

This was already constructed in the Understanding society survey dataset. The SF-12 Mental Component Summary (MCS) is a widely used measure of mental health-related quality of life derived from the 12-item Short Form Health Survey (SF-12), a condensed version of the SF-36 (Ware et al., 1996). The MCS is computed using weighted scoring algorithms that emphasise the mental health, role-emotional, social functioning, and vitality subscales while controlling for physical health components . Scores are normed to a population mean of 50 with a standard deviation of 10, where higher scores indicate better mental health functioning. The SF-12 MCS demonstrates strong psychometric properties, correlating highly (r > 0.95) with the longer SF-36 Mental Component Summary while substantially reducing respondent burden . The measure has been validated for use in British populations, with UK-specific normative data established from national surveys. The SF-12 is administered in Understanding Society (UKHLS), the UK's largest longitudinal household panel study, enabling analysis of mental health trajectories across the British population. Instruments of this scale are given here: <https://www.understandingsociety.ac.uk/documentation/mainstage/variables/sf12mcs_dv/>

**Kessler Scale**

The Kessler Psychological Distress Scale (K10) is a widely used screening instrument designed to measure non-specific psychological distress, particularly symptoms of anxiety and depression (Kessler et al., 2002). The scale comprises ten items asking respondents how often they experienced the following during the past four weeks: (1) felt tired out for no good reason, (2) felt nervous, (3) felt so nervous that nothing could calm you down, (4) felt hopeless, (5) felt restless or fidgety, (6) felt so restless that you could not sit still, (7) felt depressed, (8) felt that everything was an effort, (9) felt so sad that nothing could cheer you up, and (10) felt worthless. Responses are recorded on a five-point scale ranging from "none of the time" (scored 1) to "all of the time" (scored 5), yielding total scores from 10 to 50, with higher scores indicating greater psychological distress. The K10 has been validated for use in Australian population surveys and demonstrates strong psychometric properties, including high internal consistency and good discriminant validity for detecting DSM-IV anxiety and mood disorders. The scale is routinely administered in Australian national health surveys, including the National Health Survey and HILDA, providing comparable estimates of population mental health .

**Section 2. Robustness Analysis Graphs**

**SM Figure 1**: Mental Health Trajectories for AU over waves, when socioeconomic conditions are accounted for


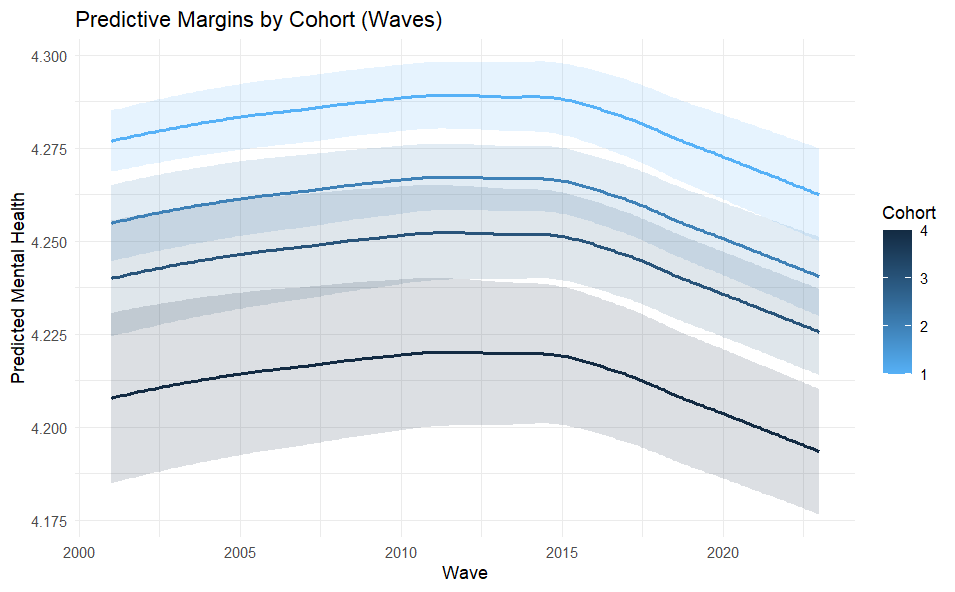


**SM Figure 2**: Mental Health Trajectories for AU over age, when socioeconomic conditions are accounted for


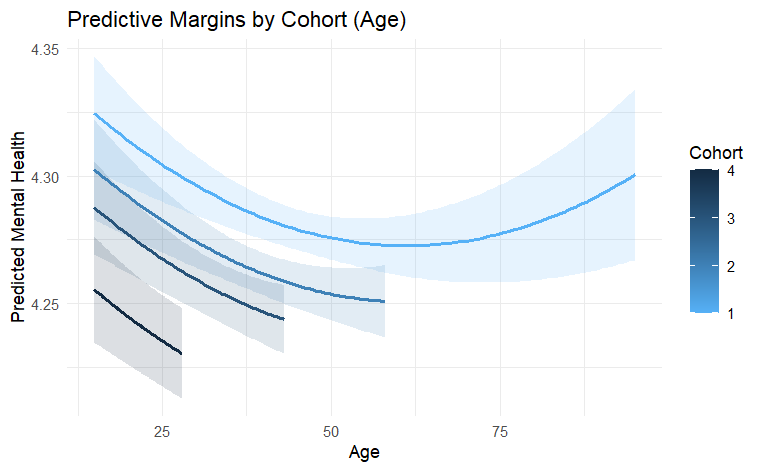


**SM Figure 3**: Mental Health Trajectories for UK over waves, when socioeconomic conditions are accounted for


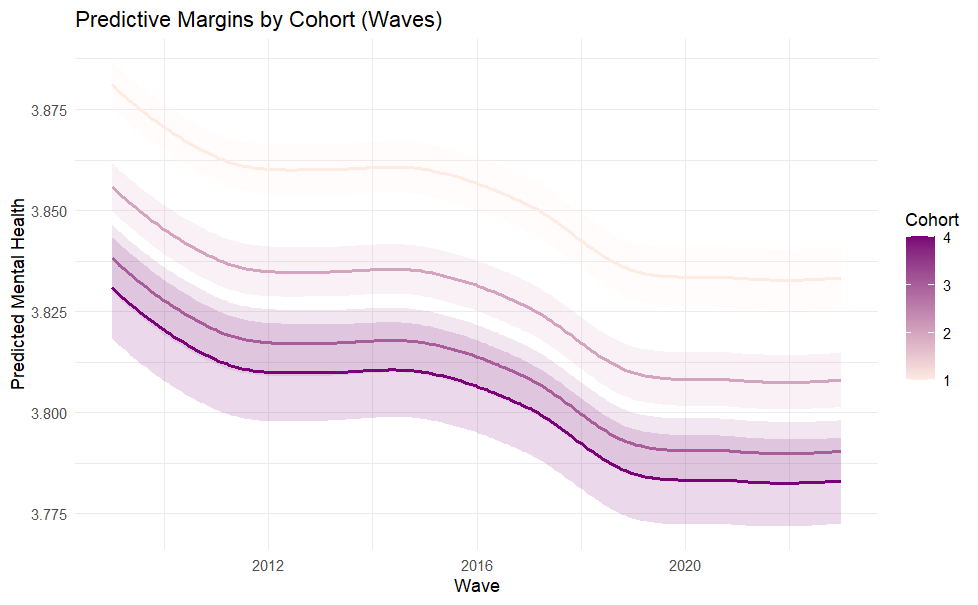


**SM Figure 4**: Mental Health Trajectories for AU over age, when socioeconomic conditions are accounted for


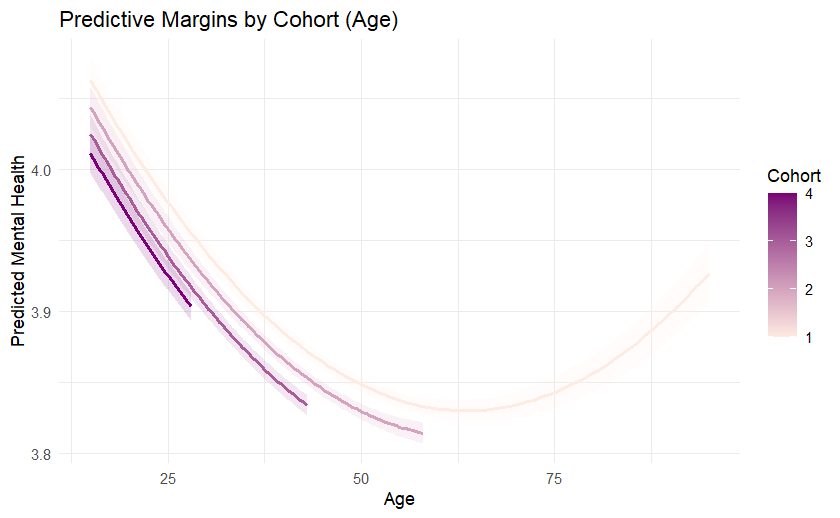


**Gender Differences**

**SM Figure 5**: Mental Health Trajectories for AU men over waves
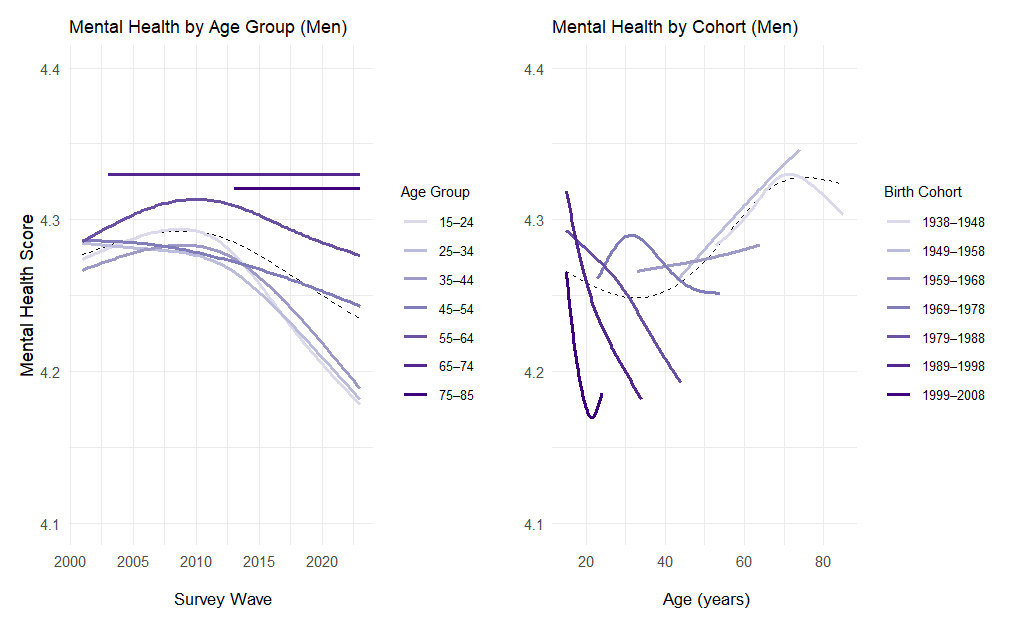


**SM Figure 6**: Mental Health Trajectories for AU women over
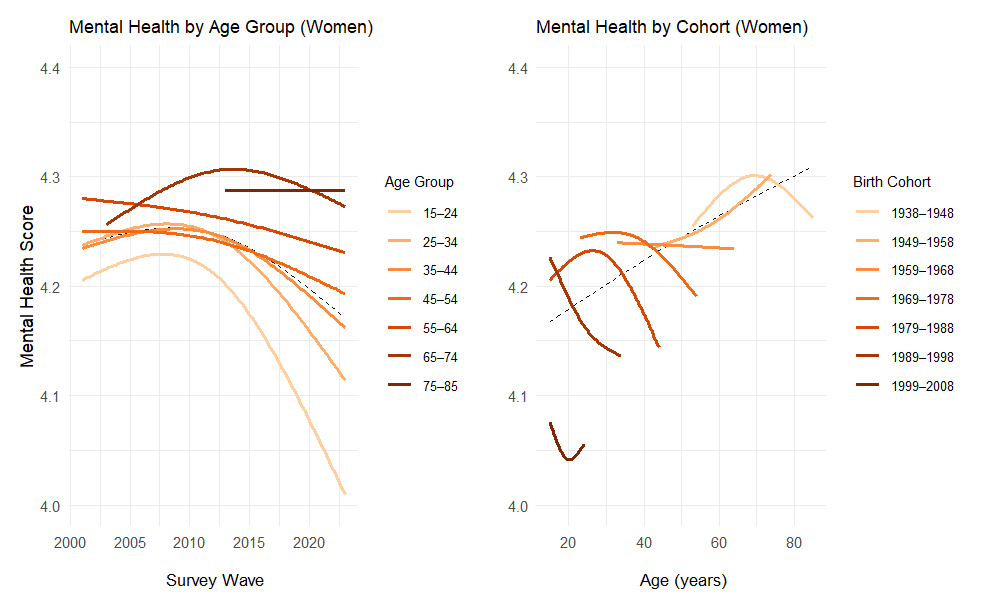
age

**SM Figure 7**: Mental Health Trajectories for UK men over waves


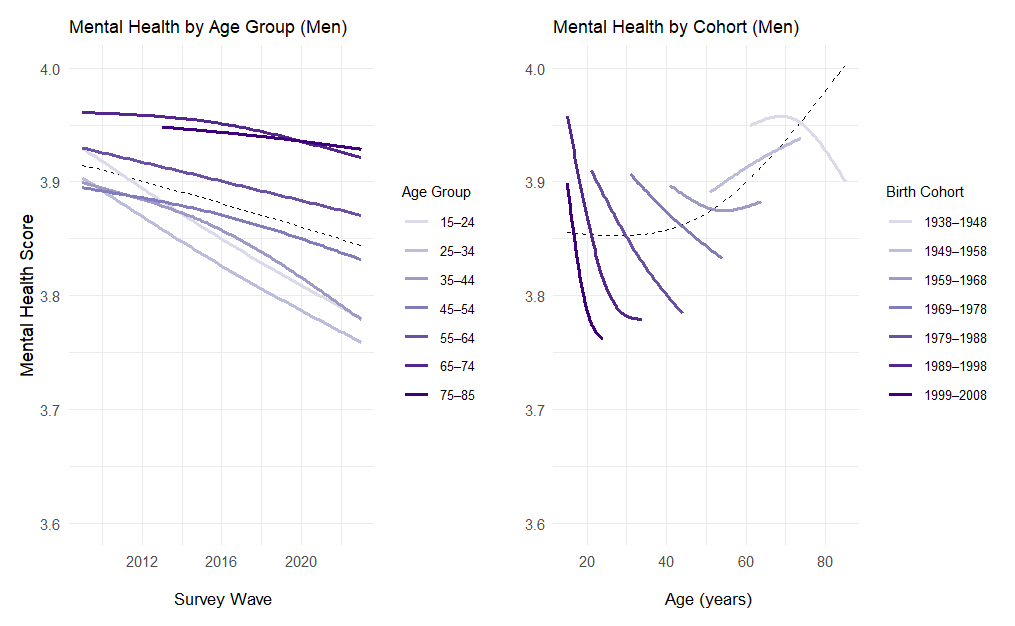


**SM Figure 8**: Mental Health Trajectories for UK women over age


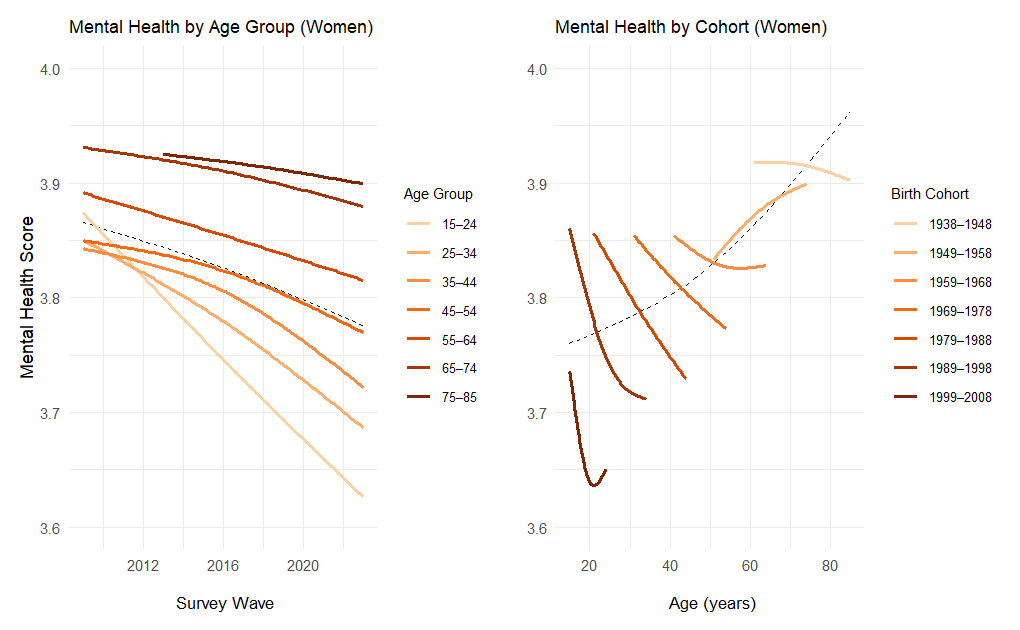


**Kessler Scale for Australia**

**SM Figure 9**: Mental Health Trajectories for AU (Kessler Scale)


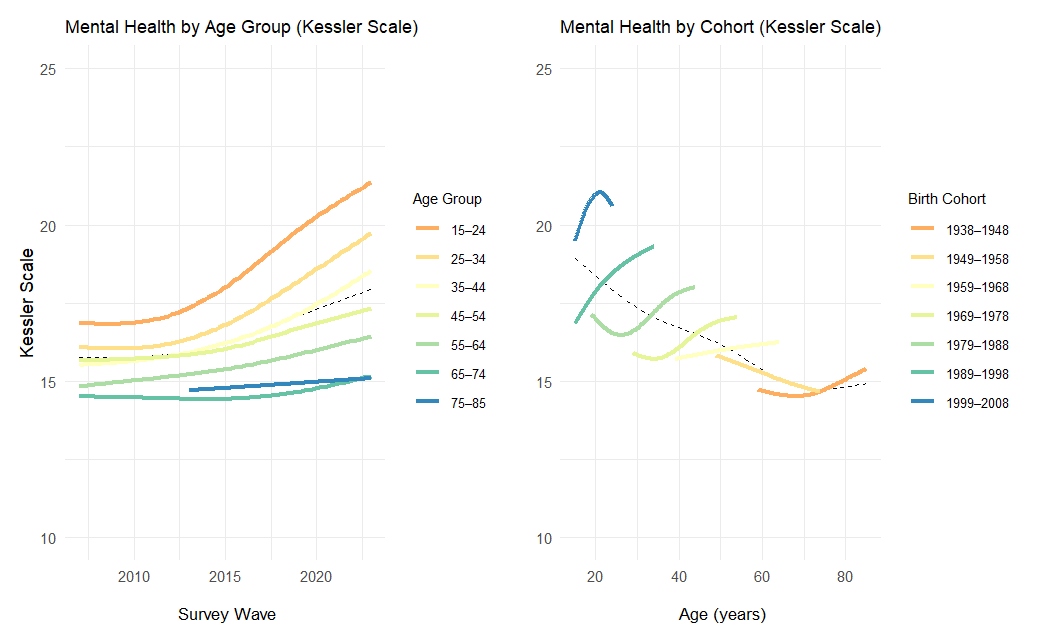


SM Figure 10. Age effect on mental health in Australia


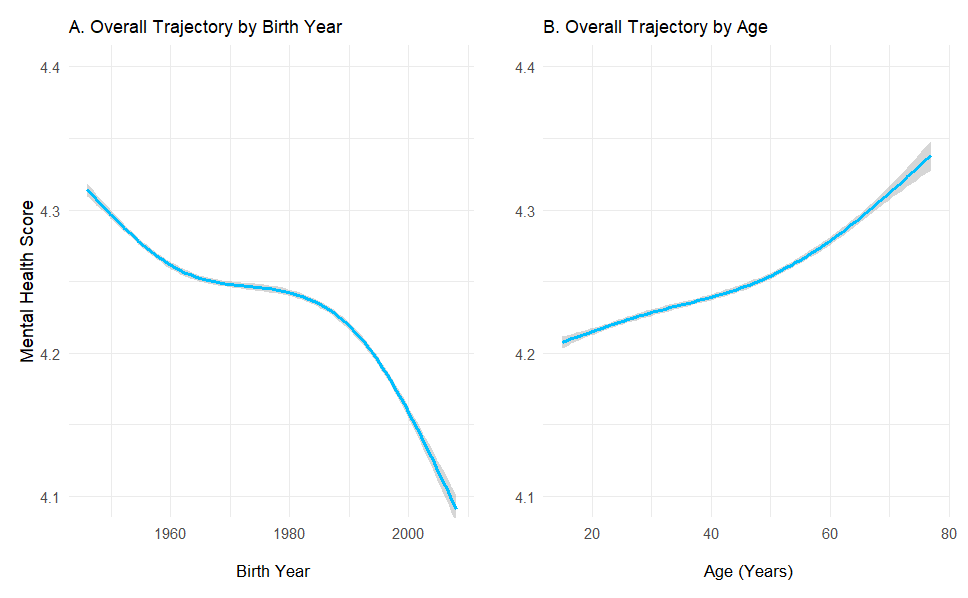


SM Figure 11. Age effect on mental health in the UK


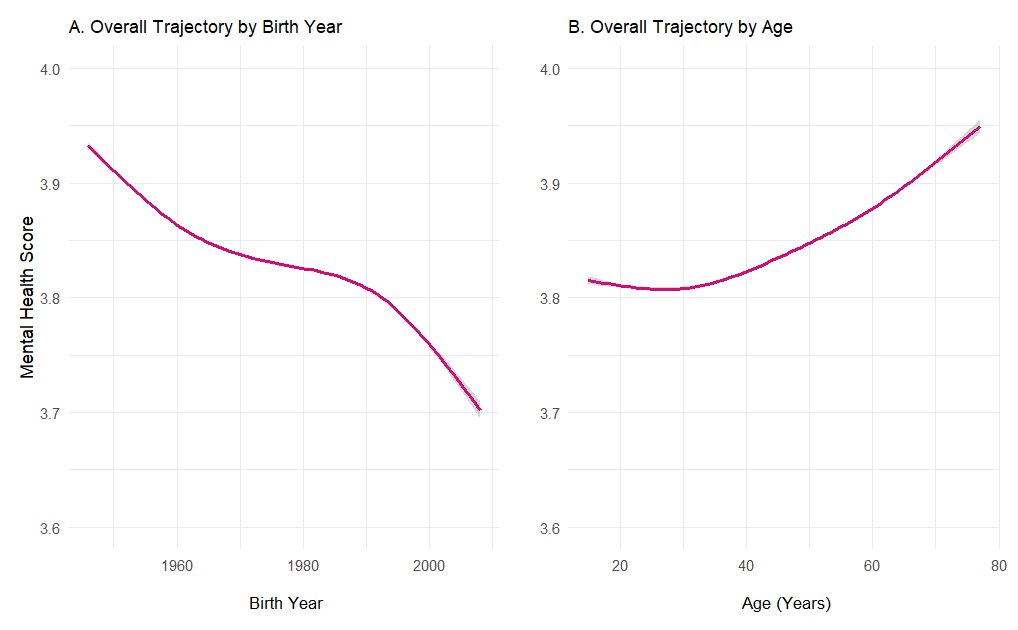

Supplement: Supplementary file 1 [file Data_Sheet_1.docx]
